# Rational Design for Multicolor Flavone-Based Fluorophores with Aggregation-Induced Emission Enhancement Characteristics and Applications in Mitochondria-Imaging

**DOI:** 10.3390/molecules23092290

**Published:** 2018-09-07

**Authors:** Liyan Liu, Yaohui Lei, Jianhui Zhang, Na Li, Fan Zhang, Huaqiao Wang, Feng He

**Affiliations:** 1 School of Pharmaceutical Science, Sun Yat-sen University, Guangzhou 510006, China; liuly37@mail2.sysu.edu.cn (L.L.); 13843159413@163.com (Y.L.); 18826072701@163.com (N.L.); zhang_fan1991@foxmail.com (F.Z.); 2 School of Chemistry, Sun Yat-sen University, Guangzhou 510275, China; zhangjh@mail.sysu.edu.cn; 3 Department of Anatomy and Neurobiology, Zhongshan School of Medicine, Sun Yat-sen University, Guangzhou 510080, China; wanghq@mail.sysu.edu.cn

**Keywords:** AIEE, multicolor, flavone, fluorophores, mitochondria

## Abstract

Fluorophores with aggregation-induced emission enhancement (AIEE) properties have attracted more attention in recent years. In order to realise more valuable applications, the different kinds of AIEE molecules are in serious need of further development. Therefore, a novel flavone-based AIEE system derived from restriction of intramolecular rotation (RIR) was designed and synthesized in this work. The results revealed that six of the compounds showed typical AIEE characteristics, with fluorescence emissions from purple, blue, cyan to green, tunable by changing substituent groups. This flavone-based AIEE system has never been reported before. The AIEE characteristics were investigated by optical spectroscopy, fluorescence photographs, scanning electron microscopy (SEM), fluorescence quantum yields (Ф_F_) and fluorescence lifetime in the CH_3_OH/H_2_O mixed solution. Moreover, benefiting from the simple structures and small molecular weight, they could permeate cells faster than current high-molecular-weight AIEE molecules. Furthermore, to examine possible biomedical applications, fluorescence imaging in living A549 lung cells and cell viabilities were examined, and the results displayed that these fluorophores showed good cellular uptake and low cytotoxicity within the experimental concentration range. In addition, these AIEE compounds possessed excellent specificity for mitochondrial targeting and mitochondrial morphological change tracking, besides, they displayed superior photostability, which indicated they are potential candidates for mitochondrial imaging.

## 1. Introduction

Fluorophores have received a great deal of attention in the areas of fundamental photophysics research and applications in biology, medicine, pharmacy and other fields [1,2,3,4]. Nevertheless, the conventional organic fluorophores frequently suffer from aggregation-caused quenching (ACQ) [5], whereby their fluorescence emission becomes weaker when the concentration of the solution increases, which limits their applications. By contrast, the novel luminescence phenomenon of aggregation-induced emission (AIE) was first discovered by Tang’s group in 2001 [6]. Inspired by Tang’s group, a lot of new AIE and AIEE molecules were reported, such as 2,20-(1,4-phenylene)-bis-(3-(4-butoxyphenyl)acrylonitrile (DBDCS), *N,N*-bis(salicylidene)-*p*-phenyl- enediamine (BSPD), distyreneanthracene (DSA) [7,8,9] and so on. Some mechanisms for the observed properties have been put forward, including restriction of intramolecular rotation (RIR), restriction of intramolecular charge transfer, *E-Z* isomerization, planarization, J-type aggregate formation and twisted intramolecular charge transfer (TICT). Among these factors RIR is considered to be the main cause [10,11,12,13,14,15,16].

**Figure 1:**
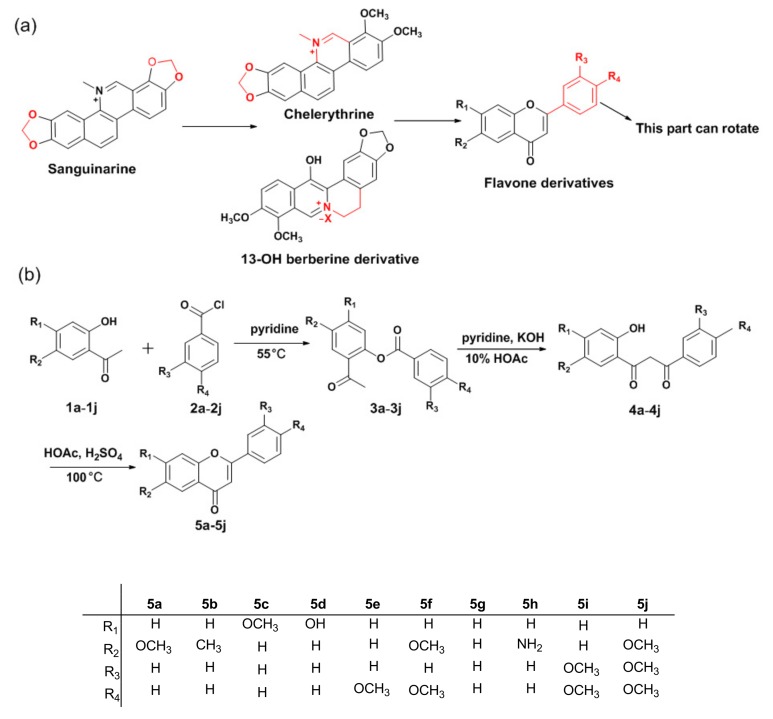
(**a**) Chemical structures of sanguinarine, chelerythrine, 13-OH berberine derivative and flavone derivatives. (**b**) Synthetic route and chemical structures of flavone derivatives.

Sanguinarine was confirmed as an AIEE molecule in our previous study [17], which is coupled with its large conjugate structure (Figure 1). According to the theory of restriction of intramolecular rotation (RIR), some primitive AIE(E) molecules were designed by increasing the number of rotatable benzene rings. These AIE(E) molecules usually have high molecular weights and bulky skeletons, including a lot of benzene rings and substituents [18,19,20], which make it difficult for the molecules to enter cells. In addition, molecules with larger rigid conjugate planes could easily become embedded in the DNA bases and result in toxicity [21], which indicated that they were mostly non-drug-like and deviated from Lipinski’s rule of 5 [22]. Conversely, in our work, reducing the conjugated structure can depress the rigidity of molecules and make a part of molecules easier to rotate, therefore, we broke the oxygen heterocyclic ring on the basis of sanguinarine, thus obtaining two new AIEE compounds, chelerythrine and a 13-OH berberine derivative, which represent novel AIEE systems different from the traditional AIEE compounds [23,24]. Based on these successful cases, a rational strategy by breaking more rings to facilitate intramolecular rotation to obtain novel AIEE systems was designed. The novel AIEE system combine small molecular weights and a certain flexibility.

Herein some novel fluorophores with AIEE characteristics are presented, as a series of flavone derivatives with hydroxy, methyl, methoxy and amino substituents were synthesized (Figure 1) and their optical properties were subsequently investigated. The results revealed that six of them (**5a**, **5b**, **5c**, **5d**, **5e** and **5f**) were typical AIEE compounds, and represent a novel AIEE system that has never been reported before. They exhibited scarce fluorescence in an extracellular solvent but became highly in fluorescent in intracellular aqueous solution, and their fluorescence emission colors upon UV irradiation were tunable from purple, blue, cyan to green by changing the substituent groups. Furthermore, these AIEE compounds not only showed very good cellular uptake and low cytotoxicity at the experimental concentration range, but also possessed excellent specificity for mitochondrial targeting and mitochondrial morphological change tracking. Furthermore, they displayed superior photostability. Some AIE(E) molecules were found to act as biological dyes and have been widely applied in morphological detection and tracking [25,26,27,28,29,30]. However, most of them suffered from monotonous color, poor photostability and the need for a high concentration to stain the target, whereas the AIEE molecules in our work avoid these limitations, which proved that they have potential for monitoring the morphological changes of mitochondria.

## 2. Results and Discussion

### 2.1. Optical Properties and Aggregation-Induced Emission Enhancement Property

**Figure 2:**
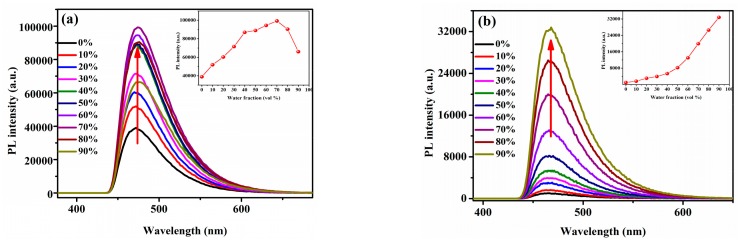

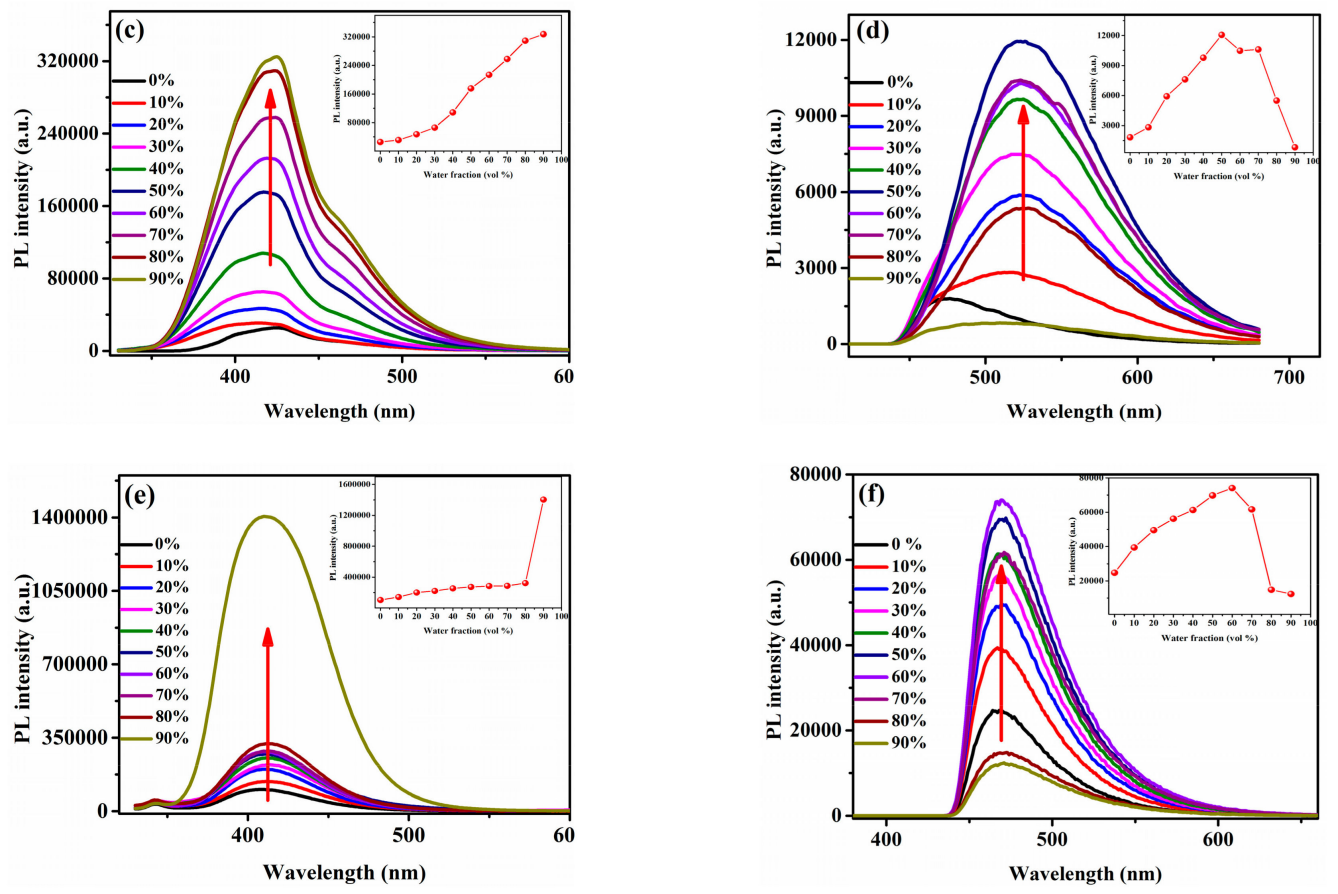
PL spectra of (**a**) **5a**, (**b**) **5b**, (**c**) **5c**, (**d**) **5d**, (**e**) **5e**, (**f**) **5f**. (c = 2.09 × 10^−5^ M) in CH_3_OH/H_2_O mixtures with different water fractions (0–90 vol%).

The fluorescence characteristics of **5a**–**j** were investigated in CH_3_OH/H_2_O mixtures (CH_3_OH as a good solvent and H_2_O as a poor solvent) where the water fraction was changed from 0% to 90%. In Figure 2b,c,e, as the water fractions changed from 0% to 90%, the PL intensity of the main emission peak increases gradually. In Figure 2b, when the water fraction is 90%, the PL intensity is enhanced 32-fold more than when it is 0%, and similar results can be found in Figure 2c, where the PL intensity is enhanced 13-fold. In Figure 2e, the PL intensity increases slowly when the fraction goes from 0% to 80%, but it suffers from a sudden increase from 80% to 90%, which is nearly 14-fold more than for 0% water. These results show that compounds **5b**, **5c** and **5e** display typical AIEE characteristics [31]. As shown in Figure 2a, the PL intensity of the main emission peak is weak in pure CH_3_OH solution and raises swiftly with the increase of water fraction, finally, reaching a maximum at 70%, which indicates a great AIEE behavior.

The PL intensity decreases when the fraction goes from 70% to 90%, which as attributed to the fact the packing mode of the molecules changes from orderly to casual [32]. The results indicate that compound **5a** is an AIEE active compound. In Figure 2d, the PL intensity is raising when the fraction changes from 0% to 50% but decreases from 50% to 90%, in Figure 2f, the PL intensity reaches a maximum when the water fraction is 60%, so we guess the attenuation of fluorescence intensity seen in Figure 2d,f is due to the same reason of Figure 2a, so in conclusion, compounds **5d** and **5f** show AIEE properties from the PL spectra. As suggested by the PL spectra in the Supporting Information (Figure S1a–d), the PL intensity of the main emission peak decreases gradually when the water fraction changes from 0% to 90% and becomes the smallest at 90%, which indicates an obvious ACQ behavior. These results reveal that compounds **5g**, **5h**, **5i** and **5j** are not active AIEE compounds.

**Figure 3:**
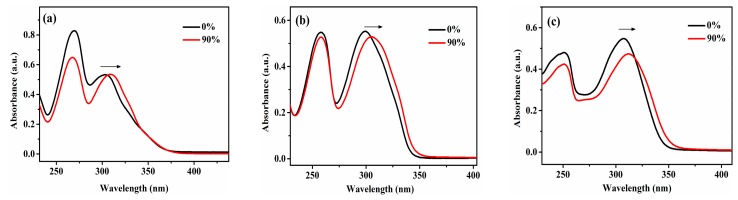

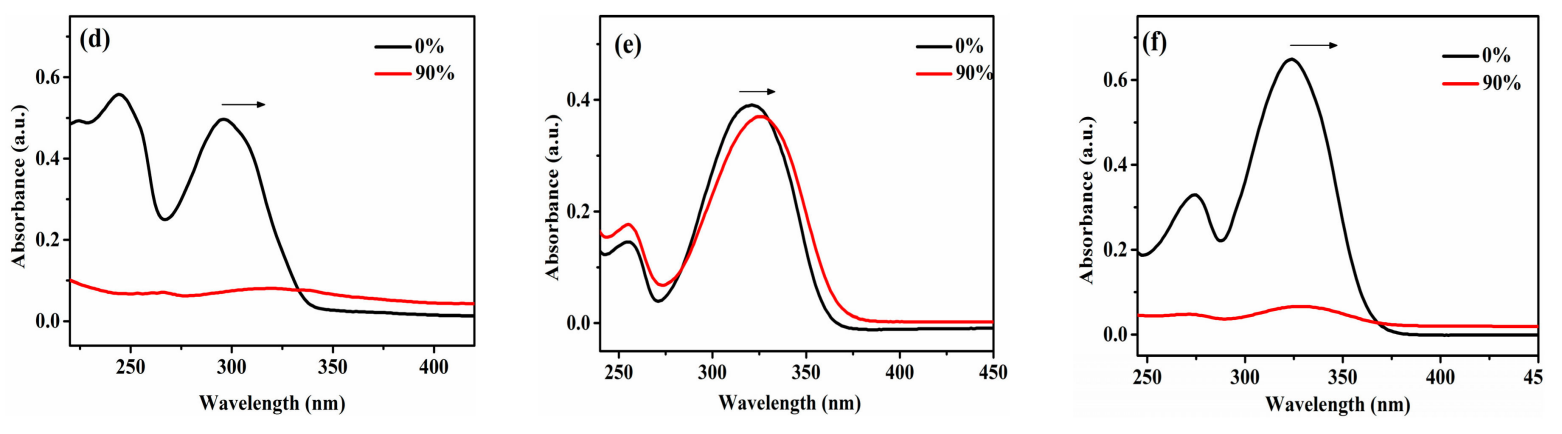
Absorption spectra of compound (**a**) **5a**, (**b**) **5b**, (**c**) **5c**, (**d**) **5d**, (**e**) **5e** and (**f**) **5f** in pure CH_3_OH solution and CH_3_OH/H_2_O (1:9, *v*/*v*) mixture (c = 2.09 × 10^−5^ M).

To further study the AIEE behavior of the compounds **5a**, **5b**, **5c**, **5d**, **5e** and **5f**, their absorption spectra were measured in CH_3_OH/H_2_O mixtures, where compounds **5a**, **5b**, **5c**, **5d**, **5e** and **5f** show absorption peaks at 302, 297, 306, 296, 320 and 324 nm in pure CH_3_OH solution, and when the water fraction changes to 90%, as shown in Figure 3, the maximum wavelengths of compounds **5a**, **5b**, **5c** and **5e** show an obvious red shift and the absorption peaks of compounds **5d** and **5f** disappear, which indicates the formation of new aggregates in the mixtures [33]. 

**Figure 4:**
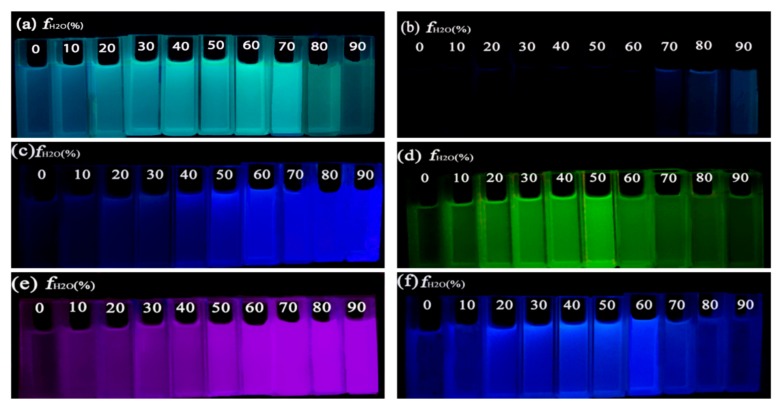
Fluorescence photographs of compounds (**a**) **5a**, (**b**) **5b**, (**c**) **5c**, (**d**) **5d**, (**e**) **5e** and (**f**) **5f** in CH_3_OH/H_2_O mixtures (with various water fractions) under a 365 nm UV excitation (c = 2.09 × 10^−5^ M).

The AIEE behavior of the compounds can also be observed from their fluorescence photographs in CH_3_OH/H_2_O mixtures with various water fractions under 365 nm UV excitation (Figure 4). We can see that compounds **5a**–**f** exhibit emissions with different colors from purple, blue, cyan to green, because the different substituent groups on the flavonoid nucleus influence the electronic density distribution of the molecules. By changing the substituents on the compounds, we can successfully achieve the regulation of the fluorescent colors of these flavone derivatives.

The fluorescence quantum yields (Ф_F_) and fluorescence lifetime of compounds **5a**, **5b**, **5c**, **5d**, **5e** and **5f** in the CH_3_OH/H_2_O mixtures were measured for a quantitative comparison. The fluorescence quantum yields (Ф_F_) were measured by using an integrating sphere. Results are displayed in Table 1, where the fluorescence quantum yields (Ф_F_) of compound **5a** in CH_3_OH, CH_3_OH/H_2_O (3:7 *v*:*v*) mixture and CH_3_OH/H_2_O (1:9 *v*:*v*) mixture are 0.16, 0.23 and 0.19, and the fluorescence lifetimes are 3.15, 3.22 and 3.19 ns, which is in accord with the behavior of the PL intensity spectrum. 

Similar phenomena occur with compounds **5b−f**, where the value of the fluorescence quantum yields (Ф_F_) and fluorescence lifetime change with the changing fluorescence intensity, which leads us to conclude that compounds **5a−f** are typical AIEE molecules.

**Figure 5:**
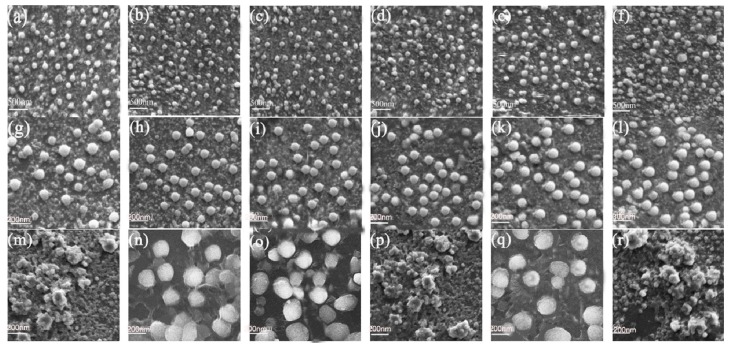
SEM images in CH_3_OH/H_2_O (5: 5 *v*:*v*) mixtures of compounds (**a**) **5a**, (**b**) **5b**, (**c**) **5c**, (**d**) **5d**, (**e**) **5e**, (**f**) **5f**. (**g**) SEM image in CH_3_OH/H_2_O (3:7 *v*:*v*) mixtures of **5a**. SEM image in CH_3_OH/H_2_O (2:8 *v*:*v*) mixtures of compounds (**h**) **5b**, (**i**) **5c**, (**k**) **5d**. SEM image in CH_3_OH/H_2_O (4:6 *v*:*v*) mixtures of (**i**) **5a**, (**l**) **5e**. SEM image in CH_3_OH/H_2_O (1:9 *v*:*v*) mixtures of (**m**) **5a**, (**n**) **5b**, (**o**) **5c**, (**p**) **5d**, (**q**) **5e**, (**r**) **5f**.

At present, there are many possible reasons that contribute to AIEE phenomena, but the RIR is identified as a main cause, so we used high viscosity (CH_3_OH/ethylene glycol) solvents and SEM to confirm the AIEE mechanism. As the proportion of ethylene glycol increased, the viscosity of the solutions increased, which restricts the rotation of the molecules and leads to a fluorescence enhancement [34]. This speculation is supported by the fluorescence spectra in Figure S2, where the emission of compounds **5a**–**f** increases significantly in pace with the increase of viscosity, reaching a maximum when the ethylene glycol fraction is 50%. The SEM results shown in Figure 5 provide plentiful information to understand the reason for the AIEE effect, as many nanoparticle aggregates with approximately spherically shapes are formed and, as can be observed from the images, the shape of the nanoparticles remains spherical but their diameter increases with the increase of water content, so we can conclude that the formation of large diameter spherical nanoparticles leads to a fluorescence enhancement in the CH_3_OH/H_2_O mixtures. Meanwhile, the shape of nanoparticles changes when the water fraction is 90%, which indicates that they aggregate into different shapes and new aggregates are formed [35]. These results correspond to the emission decrease of **5a**, **5f** and **5d** at 90% water fraction. Time-dependent fluorescence spectra of **5a**–**f** are shown in Figure S3, where there are no obvious changes in fluorescence intensity with increasing irradiation time, showing that compounds **5a**–**f** are stable in CH_3_OH/H_2_O mixtures, a property that is convenient for their application in other areas.

### 2.2. Electronic Structure

**Figure 6:**
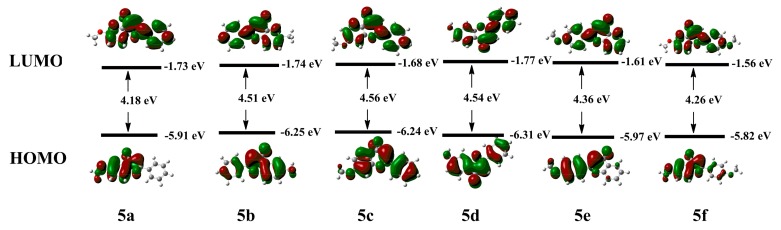
Frontier molecular orbital amplitude plots and energy levels of the HOMOs and the LUMOs of **5a**–**f**.

In order to better understand their optical properties, the frontier molecular orbitals and energy levels of the highest occupied molecular orbitals (HOMOs) and the lowest unoccupied molecular orbitals (LUMOs) of **5a**–**f** were calculated by density functional theory using the Gaussian 09 program. In Figure 6, the electron density of the HOMOs of **5b**, **5c**, **5d** are mainly localized on the whole skeleton of the molecules and in **5a**, **5e**, **5f** they are mainly localized on the A and C rings of the molecules, while the electron density of the LUMOs of **5a**–**f** are mainly localized on the whole skeleton of the molecules, and the phase positions of the HOMOs and LUMOs are basically symmetrical, so electrons can easily flow from HOMOs to LUMOs, which illustrates these molecules have a good potential for producing fluorescence. The energy gaps between the HOMOs and LUMOs for **5a**–**f** are 4.18, 4.51, 4.56, 4.54, 4.36 and 4.26 eV, respectively. These small energy gaps enable a facile electron transit, which indicates these molecules can be excited easily. The results are well consistent with the strong fluorescence emission of compounds **5a**–**f**. In addition, the small energy gap can result in a red-shift, which agrees very well with the absorption spectra measurement results shown in Figure 3.

### 2.3. Fluorescence Imaging in Living Cells and Cell Viability Assay

**Figure 7:**
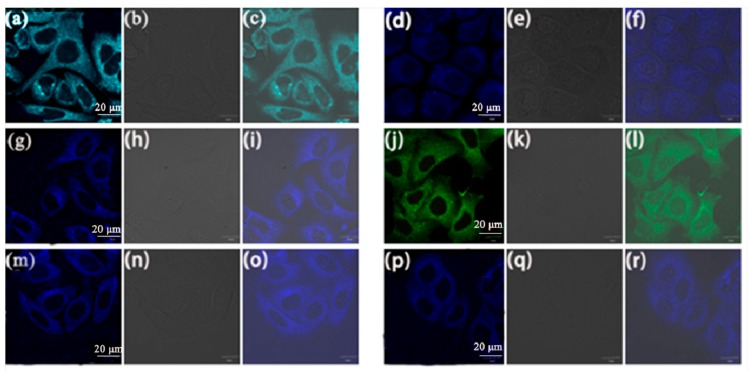
CLSM images of A549 cells incubated with (**a**) **5a**, (**d**) **5b**, (**g**) **5c**, (**j**) **5d**, (**m**) **5e** and (**p**) **5f** (10 μM) for 30 min with excitation at 405 nm; The bright-field image of A549 cells stained with (**b**) **5a**, (**e**) **5b**, (**h**) **5c**, (**k**) **5d**, (**n**) **5e**, (**q**) **5f**. The merged image stained with (**c**) **5a**, (**f**) **5b**, (**i**) **5c**, (**l**) **5d**, (**o**) **5e**, (**r**) **5f**. Scale bar: 20 μm.

To study the applications of compounds **5a**–**f** for biological imaging, cell uptake experiments were performed with A549 cancer cells. The A549 cells were cultured separately at 37 °C for 30 min with **5a**–**f** (at 10 μM concentration). As shown in Figure 7, bright fluorescence with the color of blue, cyan and green is observed in the cytoplasm of the A549 cells from the CLSM images. These compounds are therefore cell permeable and display strong fluorescence. The bright fluorescence of **5b**–**f** is blue, that of **5a** is cyan and it is green for **5d**. The stronger fluorescence signals in the CLSM images indicate that these AIEE flavone derivatives aggregate in intracellular aqueous solution and show very good cellular uptake.

**Figure 8:**
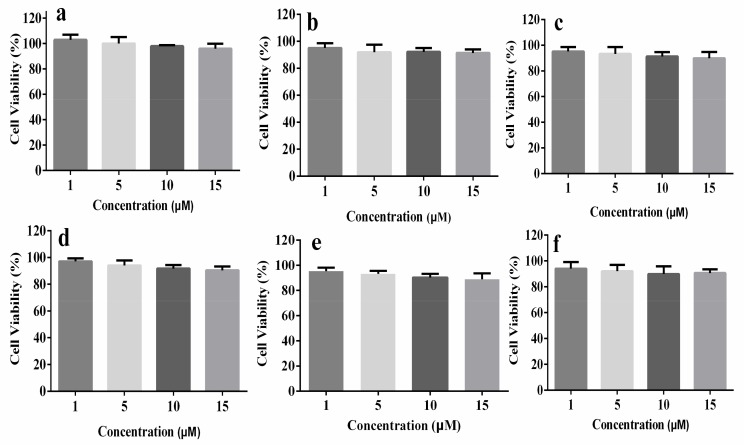
Cell viabilities of A549 cells treated with different concentrations of (**a**) **5a**, (**b**) **5b**, (**c**) **5c**, (**d**) **5d**, (**e**) **5e**, (**f**) **5f** for 24 h by MTT assay.

Cell viability, which decides the potential for biological imaging, is an important factor for the further study of biological applications, so the cytotoxicity of compounds **5a**–**f** in A549 cells was investigated by using a MTT assay. The A549 cells were cultured at 37 °C with different concentrations (1, 5, 10, 15 μM) of compounds **5a**–**f** for 24 h. The results shown in Figure 8 reveal that these compounds show low cytotoxicity in the experimental concentration range, and at a concentration of 10 μM, the cell viability values of all the compounds are more than 90%, which suggests that compounds **5a**–**f** have high potential for live cell imaging applications.

### 2.4. Mitochondrial Imaging

The fluorescence imaging of compounds **5a**, **5b**, **5c**, **5d**, **5e** and **5f** in Figure 7 indicates that these compounds can enter the cell membrane and gather in some specific parts of the cell, thus, co-localization experiments with Mito Tracker Deep Red were performed in order to confirm the specific location of the aggregation. The A549 cells were separately cultured at 37 °C for 30 min with Mito Tracker Deep Red (100 nM) and **5a**–**f** (10 μM).

**Figure 9:**
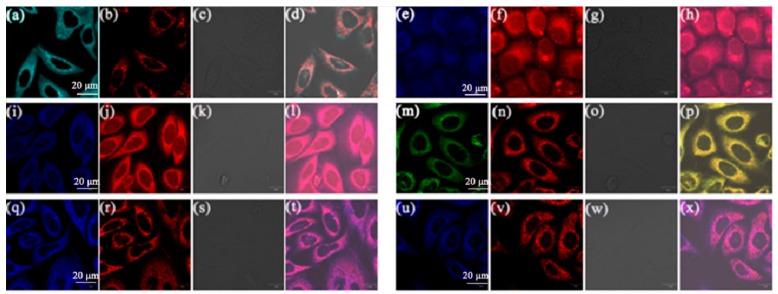
Co-localized images of A549 cells incubated with (**a**) **5a**, (**e**) **5b**, (**i**) **5c**, (**m**) **5d**, (**q**) **5e** and (**u**) **5f** (10 μM) for 30 min and Mito Tracker Red (200 nM) (**b**) **5a**, (**f**) **5b**, (**j**) **5c**, (**n**) **5d**, (**r**) **5e**, (**v**) **5f** for 15 min with excitation at 405 nm; The bright-field image of A549 cells stained with (**c**) **5a**, (**g**) **5b**, (**k**) **5c**, (**o**) **5d**, (**s**) **5e**, (**w**) **5f**. The merged image stained with (**d**) **5a**, (**h**) **5b**, (**l**) **5c**, (**p**) **5d**, (**t**) **5e**, (**x**) **5f**. Scale bar: 20 μm.

As displayed in Figure 9, these compounds stain the mitochondrial region specifically in A549 cells, and the fluorescence signals with different colors from compounds **5a**–**f** are overlapped well with the red fluorescence signals from Mito Tracker by colocalization analysis [36]. The reticulum structures of the mitochondria are clearly visible under the fluorescence of compounds **5a**–**f**, which indicates that the fluorescence from these compounds is localized on the mitochondria of the living A549 cells. The Pearson’s correlation coefficient (Rr; from +1 to −1) which indicates the linear correlation between two variables, is used to quantify the staining region overlap between compounds **5a**–**f** and MT. The coefficients are 0.96, 0.95, 0.95, 0.97, 0.98 and 0.95 for **5a**–**f**, respectively (Figure S4), indicating the specific targeting ability of these AIEE active flavone derivatives on mitochondria.

### 2.5. Mitochondrial Morphological Change

**Figure 10:**
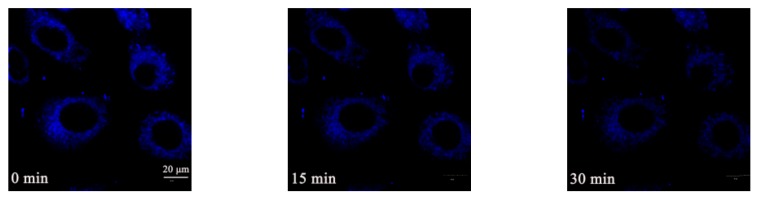
CLSM images of A549 cells treated with CCCP (20 μM) stained with **5e** (10 μM) with increasing scanning time (0, 15, 30 min). Scale bar: 20 μm.

According to the literature, mitochondria continuously oxidize substrates and preserve a proton gradient across the phospholipid bimolecular layer with a large membrane potential (Δ*Ψ*_m_) of around −180 mV [37]. To further research the ability of these AIEE flavone derivatives, morphological change tracking experiments were carried out. Carbonyl cyanide *m*-chlorophenylhydrazone (CCCP), which is known as an uncoupler that causes the quick acidification of mitochondria and malfunction of ATP synthase, thus bringing about the decrease of the mitochondrial Δ*Ψ*_m_ [38], was used to treat the living A549 cells before staining. Compound **5e** was selected as a representative of these active AIEE flavone derivatives for the staining experiments because of its strong fluorescence emission. As shown in Figure 10, after staining by CCCP, the reticulum-like mitochondria were transformed into small and dispersed fragments step by step, which was observed clearly with the blue fluorescence of **5e**. Compound **5e** showed outstanding photostability during this process. Mitochondrial morphological changes are involved in the early stages of apoptosis and are considered an irreversible process, so **5e** has potential for apoptosis research. In conclusion, compound **5e** showed excellent ability for tracking mitochondrial morphological changes, which suggested these active AIEE flavone derivatives are potential candidates for mitochondrial research.

## 3. Materials and Methods

### 3.1. Materials and Instruments

All the regents and analytical grade solvents in this paper were obtained commercially and used without further purification unless indicated. 2′-Hydroxyacetophenone, benzoyl chloride, 2′-hydroxy-5′-methoxyacetophenone, 2′-hydroxy-4′-methoxyacetophenone and 2′,4′-dihydroxy- acetophenone were obtained from Macklin (Shanghai, China). 3,4-Dimethoxybenzoylchoride, 4-methoxybenzoylchoride and 2′-hydroxy-5′-methylacetophenone were obtained from Sigma-Aldrich (Shanghai, China), Pyridine and potassium hydroxide (KOH) were from Aladdin (Shanghai, China). Pyridine was distilled from anhydrous potassium hydroxide (KOH) before use. ^1^H-NMR (400 MHz) and ^13^C-NMR (101 MHz) spectra were obtained on an Avance III 400 MHz spectrometer (Bruker, Karlsruhe, Germany) in CDCl_3_. The progress of reactions was checked by analytical thin layer chromatography (TLC). The mass spectra were obtained on a LCMS-IT-TOF mass spectrometer (Shimadzu, kyoto, Japan). Photoluminescence (PL) spectra and absolute PL quantum yields were obtianed on an FLS920 spectrophotometer (Edinburgh Instruments, Edinburgh, UK). The fluorescence lifetimes were obtained with an FLS 980 spectrometer (Edinburgh Instruments, Edinburgh, UK). Ultraviolet (UV) absorption spectra were collected on a UV-2600 spectrometer (Shimadzu, Kyoto, Japan). Scanning electron microscope (SEM) images were obtained on a Zeiss Merlin emission scanning electron microscope (Zeiss Co., Oberkochen, Germany). The human lung cancer A549 cell line was obtained from laboratory animal center of Sun Yat-sen University. Commercially available RPMI Medium Modified, Dulbecco’s modified Eagle’s Medium (DMEM), 0.25% trypsin solution, phosphate buffered saline (PBS), the Mito Tracker Red assay kit (MT) and 3-(4,5-dimethyl-2-thiazolyl)-2,5-diphenyl-2-*H*-tetrazolium bromide (MTT) were obtained from Thermo-Fisher Biochemical Products (Beijing, China). Fluorescent images were examined on an OLYMPUS FV3000 laser scanning confocal microscope (Zeiss Co., Oberkochen, Germany) and cell viabilities were analyzed by a Flex Station 3 microplate reader (Molecular Devices, silicon valley, USA).

### 3.2. Synthesis of Flavone Derivatives

The structures and synthesis route of the flavone derivatives are exhibited in Figure 1. This route involves three steps. The reaction involved in the first step was a modified Schotten-Baumann reaction, then, a Baker-Venkataraman rearrangement and cyclization catalyzed by acid were involved in the next steps [39,40]. The final products **5a**–**j** were obtained in good yield.

#### 3.2.1. Synthesis of Compounds **3a**–**j**

Compounds **1a**–**j** (0.9 mL), compounds **2a**–**j** (1 mL) and dried and distilled pyridine (2.5 mL) were put into in a 50 mL round bottomed flask equipped with a reflux condenser, then the mixture was stirred about 30 min at 55 °C, and then poured into hydrochloric acid (60 mL, 1 mol/L) mixed with 25 g of crushed ice, then stirred until solids were formed. After that the solids were filtered from the solution and washed by 2.5 mL methanol and 2.5 mL water, to give, after recrystallization from methanol and water, compounds **3a**–**j**.

#### 3.2.2. Synthesis of Compounds **4a**–**j**

Compounds **3a**–**j** (0.01 mol), dried and distilled pyridine (9 mL) and powdered KOH (0.03 mol) were placed a round bottomed flask equipped with a reflux condenser and stirred for about 15 min at 50 °C. After the reaction, the mixture was allowed to cool to room temperature, then, aqueous acetic acid solution (12.5 mL, 10% concentration) were added into the mixture, giving compounds **4a**–**j** after the solids were formed and filtered.

#### 3.2.3. Synthesis of Compounds **5a**–**j**

Compounds **4a**–**j** (1.8 g) glycerol triacetate (10 mL) and concentrated sulfuric acid (0.4 mL) were placed in a round bottomed flask equipped with a reflux condenser, stirred about 1 h at 100 °C and then the mixture was poured into a beaker containing 50 g of crushed ice, where solids were formed after stirring and then filtered from the solution, Next the solids were washed with water until the acid was removed. Finally, the solids were purified and separated by column chromatography eluting with a mixed petroleum ether and ethyl acetate solvent until the final products **5a**–**j** were obtained.

*6-Methoxyflavone* (**5a**). Yield: 78%; white solid, mp.: 163–165 °C; ^1^H-NMR (CDCl_3_) δ 7.93–7.80 (m, 1H), 7.52 (t, *J* = 7.6 Hz, 2H), 7.51–7.39 (m, 1H), 7.24 (d, *J* = 3.1 Hz, 1H), 7.22 (d, *J* = 3.1 Hz, 2H), 7.19 (s, 1H), 6.79 (s, 1H), 3.85 (s, 3H); ^13^C-NMR (CDCl_3_) δ 177.27, 162.12, 155.98, 150.04, 130.83, 130.46, 127.99, 127.99, 125.20, 125.20, 123.52, 122.76, 118.48, 105.79, 103.81, 54.90. MS (ESI-MS) *m*/*z* calcd for C_16_H_12_O_3_ [M + H]^+^: 253.26; found: 253.28.

*6-Methylflavone* (**5b**). Yield: 74%; white solid, mp.: 119–122 °C; ^1^H-NMR (CDCl_3_) δ 7.96 (s, 1H), 7.90–7.83 (m, 1H), 7.46 (dd, *J* = 5.1, 2.2 Hz,2H), 7.44 (d, *J* = 1.9 Hz, 1H), 7.42 (s, 1H), 7.40 (s, 1H), 7.19 (s, 1H), 6.79 (s, 1H), 2.41 (s, 3H); ^13^C-NMR (CDCl_3_) δ 177.54, 162.25, 153.53, 134.19, 133.97, 130.88, 130.48, 127.99, 127.99, 125.24, 125.24, 124.03, 122.58, 116.82, 106.40, 19.91. MS (ESI-MS) *m*/*z* calcd for C_16_H_12_O_2_ [M + H]^+^: 237.26; found: 237.27.

*7-Methoxyflavone* (**5c**). Yield: 57%; slight yellow solid, mp.: 110–112 °C; ^1^H-NMR (CDCl_3_) δ 8.08 (dd, *J* = 8.8, 3.5 Hz, 1H), 7.90–7.78 (m, 1H), 7.60–7.53 (m, 1H), 7.48–7.48 (m, 1H), 7.49–7.46 (m, 1H), 7.46–7.43 (m, 1H), 7.37–7.24 (m, 1H), 6.99–6.88 (m, 1H), 6.79 (s, 1H), 3.87 (s, 3H); ^13^C-NMR (CDCl_3_) δ 176.91, 163.20, 162.04, 157.01, 130.84, 130.41, 128.35, 128.35, 126.04, 126.04, 125.16, 116.81, 113.43, 106.53, 99.42, 54.85. MS (ESI-MS) *m*/*z* calcd for C_16_H_12_O_3_ [M + H]^+^: 253.26; found: 253.24.

*7-Hydroxyflavone* (**5d**) Yield: 59%; slight yellow solid, mp.: 244–246 °C; ^1^H-NMR (CDCl_3_) δ 8.20 (dd, *J* = 29.6, 8.2 Hz, 3H), 7.89–7.58 (m, 3H), 7.48 (s, 1H), 7.24 (dd, *J* = 8.6, 1.7 Hz, 1H), 6.78 (s, 1H); ^13^C-NMR (CDCl_3_) δ 176.76, 163.39, 162.76, 155.79, 153.95, 133.12, 130.64, 129.32, 128.09, 127.76, 126.19, 125.30, 118.58, 110.29, 106.71; MS (ESI-MS) *m*/*z* calcd for C_15_H_10_O_3_ [M + H]^+^: 239.12; found: 239.16.

*4′-Methoxyflavone* (**5e**). Yield: 71%; white solid, mp.: 161–163 °C; ^1^H-NMR (CDCl_3_) δ 8.23 (d, *J* = 7.9 Hz, 1H), 7.90 (d, *J* = 8.8 Hz, 2H), 7.69 (t, *J* = 7.7 Hz, 1H), 7.56 (d, *J* = 8.4 Hz, 1H), 7.42 (t, *J* = 7.5 Hz, 1H), 7.03 (d, *J* = 8.8 Hz, 2H), 6.77 (s, 1H), 3.90 (s, 3H); ^13^C-NMR (CDCl_3_) δ 178.37, 163.48, 162.46, 156.21, 133.60, 128.04, 128.04, 125.68, 125.11, 124.05, 123.92, 117.97, 114.49, 114.49, 106.17, 55.52; MS (ESI-MS) *m*/*z* calcd for C_16_H_12_O_3_ [M + H]^+^: 253.26; found: 253.28.

*6,4′-Methoxyflavone* (**5f**). Yield: 75%; white solid, mp.: 185–188 °C; ^1^H-NMR (CDCl_3_) δ 7.88–7.76 (m, 1H), 7.53 (d, *J* = 3.1 Hz, 2H), 7.43 (d, *J* = 9.1 Hz, 1H), 7.23 (d, *J* = 3.1 Hz, 1H), 7.21–7.19 (m, 1H), 7.01–6.91 (m, 1H), 6.73 (s, 1H), 3.83 (d, *J* = 8.3 Hz, 6H); ^13^C-NMR (CDCl_3_) δ 178.24, 163.21, 162.33, 156.91, 150.99, 127.93, 127.93, 124.52, 124.11, 123.53, 119.38, 114.44, 114.44, 105.47, 104.89, 55.93, 55.50; MS (ESI-MS) *m*/*z* calcd for C_17_H_14_O_4_ [M + H]^+^: 283.29; found: 283.21.

*Flavone* (**5g**). Yield: 81%; white solid, mp: 95–97 °C; ^1^H-NMR (CDCl_3_) δ 8.25(d, *J* = 7.9 Hz, 1H), 7.99–7.90 (m, 2H), 7.71 (t, *J* = 7.8 Hz, 1H), 7.63–7.49 (m, 4H), 7.43 (t, *J* = 7.5 Hz, 1H), 6.84 (s, 1H); ^13^C-NMR (CDCl_3_) δ 177.40, 162.41, 155.25, 132.76, 130.76, 130.68, 128.03, 128.03, 125.28, 124.69, 124.22, 124.22, 122.93, 117.07, 106.56. MS (ESI-MS) *m*/*z* calcd for C_15_H_10_O_2_ [M + H]^+^: 223.24; found: 223.12.

*6-Aminoflavone* (**5h**). Yield: 66%; slight yellow solid, mp: 196–200 °C; ^1^H-NMR (CDCl_3_) δ 7.87–7.84 (m, 1H), 7.83 (dd, *J* = 6.3, 3.1 Hz, 1H), 7.45 (d, *J* = 2.0 Hz, 1H), 7.43 (dd, *J* = 7.3, 3.2 Hz, 1H), 7.36–7.33 (m, 1H), 7.33 (s, 1H), 7.00 (d, *J* = 2.8 Hz, 1H), 6.97 (d, *J* = 2.9 Hz, 1H), 6.70 (s, 1H), 3.83 (s, 2H); ^13^C-NMR (CDCl_3_) δ 177.48, 161.99, 148.95, 143.10, 131.09, 130.31, 127.95, 127.95, 125.19, 125.19, 123.72, 121.21, 118.04, 106.91, 105.67; MS (ESI-MS) *m*/*z* calcd for C_15_H_11_O_2_N [M + H]^+^: 238.25; found: 238.27.

*3′,4′-Methoxyflavone* (**5i**). Yield: 72%; white solid, mp: 154–155 °C; ^1^H-NMR (CDCl_3_) δ 8.17 (dd, *J* = 7.9, 1.6 Hz, 1H), 7.69–7.59 (m, 1H), 7.52 (dd, *J* = 8.3, 3.0 Hz, 2H), 7.37 (d, *J* = 7.2 Hz, 1H), 7.34 (d, *J* = 2.1 Hz, 1H), 6.93 (d, *J* = 8.5 Hz, 1H), 6.77 (s, 1H), 3.93 (s, 3H), 3.91 (s, 3H); ^13^C-NMR (CDCl_3_) δ 177.34, 162.35, 155.16, 151.08, 148.28, 132.5, 124.64, 124.12, 123.22, 122.92, 119.00, 116.96, 110.16, 107.85, 105.45, 55.07, 55.07; MS (ESI-MS) *m*/*z* calcd for C_17_H_14_O_4_ [M + H]^+^: 283.29; found: 283.19.

*6,3′,4′-Methoxyflavone* (**5j**). Yield: 69%; white solid, mp.: 183–185 °C; ^1^H-NMR (CDCl_3_) δ 7.53 (dd, *J* = 6.3, 2.6 Hz, 1H), 7.50 (d, *J* = 2.1 Hz, 1H), 7.46 (d, *J* = 9.1 Hz, 1H), 7.33 (d, *J* = 2.1 Hz, 1H), 7.23 (dd, *J* = 9.1, 3.1 Hz, 1H), 6.92 (d, *J* = 8.5 Hz, 1H), 6.79 (s, 1H), 3.92 (s, 3H), 3.90 (s, 3H), 3.85 (s, 3H); ^13^C-NMR (CDCl_3_) δ 178.20, 163.20, 156.97, 152.03, 151.00, 149.29, 124.42, 123.61, 123.61, 119.95, 119.40, 111.18, 108.84, 105.76, 104.87, 56.09, 55.94, 55.94; MS (ESI-MS) *m*/*z* calcd for C_18_H_16_O_5_ [M + H]^+^: 313.32; found: 313.33.

### 3.3. Preparation for UV–Vis Spectra, Pl Spectra and SEM Measurements

The flavone derivatives were prepared as a series of stock methanol solutions with a concentration of 10^−4^ M. Then different volumes of methanol and water were added cautiously under vigorous stirring, the water fractions changed from 0 to 90 vol% while the final concentration was kept at 10^−5^ M. UV-vis absorption spectra and PL spectra compounds **5a**–**j** were measured immediately at room temperature with the excitation wavelengths 360, 346, 305, 396, 310, 360, 310, 354, 365, 363 nm respectively in the CH_3_OH/H_2_O mixtures. The absolute PL quantum yields (Ф_F_) of **5a** in CH_3_OH, CH_3_OH/H_2_O (3:7, *v*/*v*) mixture, CH_3_OH/H_2_O (1:9, *v*/*v*) mixture, **5b** in CH_3_OH, CH_3_OH/H_2_O (5:5, *v*/*v*) mixture, CH_3_OH/H_2_O (1:9, *v*/*v*) mixture, **5c** in CH_3_OH, CH_3_OH/H_2_O (5:5, *v*/*v*) mixture, CH_3_OH/H_2_O (1:9, *v*/*v*) mixture, **5d** in CH_3_OH, CH_3_OH/H_2_O (5:5, *v*/*v*) mixture, CH_3_OH/H_2_O (1:9, *v*/*v*) mixture, **5e** in CH_3_OH, CH_3_OH/H_2_O (5:5, *v*/*v*) mixture, CH_3_OH/H_2_O (1:9, *v*/*v*) mixture and **5f** in CH_3_OH, CH_3_OH/H_2_O (4:6,*v*/*v*) mixture, CH_3_OH/H_2_O(1:9, *v*/*v*) mixture were recorded on an integrating sphere and the fluorescence lifetimes of **5a**–**f** were obtained on a FLS 980 spectrometer with the same water fractions change as the absolute PL quantum yields. Solutions of **5a**–**f** with the same water fraction change of fluorescence lifetime were placed dropwise on a silicon wafer respectively and evaporated over 12 h at room temperature. After completely drying, metal spraying was performed for SEM imaging [41,42].

### 3.4. Preparation for Ethylene Glycol (EG) Measurement

The flavone derivatives were dissolved in methanol solutions with a concentration of 10^−4^ M. Then different volumes of methanol and EG were added cautiously with the EG fractions changed from 0 to 50 vol% and the final concentration was kept at 10^−5^ M. As the EG fractions changed the viscosity of the solution changed [43]. After stirring, PL spectra of **5a**–**f** with different EG fractions were measured immediately at room temperature using a FLS 980 spectrometer.

### 3.5. Theoretical Calculations

The highest occupied molecular orbital (HOMO) and the lowest unoccupied molecular orbital (LUMO) of **5a**–**f** were calculated by density functional theory (DFT) using the Gaussian 09 program at the B3LYP/6-31G* level.

### 3.6. Cell Viability Evaluation

A549 lung cancer cells were seeded in 96-well microplates at a density of 9 × 10^3^ cells per well and maintained overnight in RPMI Medium Modified (RPMI-1640) media, containing 10% fetal bovine serum (FBS), 10 mg/mL of streptomycin and 10 mg/mL penicillin. After 24 h of incubation in an incubator with 5% CO_2_ at 37 °C, treated with various concentrations of flavone derivatives (1−15 μM) at 37 °C for 24 h. Then the medium was poured out and 20 μL 0.5% MTT (1 mg/mL in phosphate buffered saline [PBS]) was added to each well, then, incubated for 4 h at 37 °C. After 4 h the medium was removed, 150 μL DMSO was added to per well and shaken slowly for 10 min. Absorbance of plates were then monitored by a microplate reader (Molecular Devices) at 490 nm.

### 3.7. Cell Imaging

A549 cells were cultured in RPMI-1640 medium containing 10% fetal bovine serum (FBS), then seeded in a glass bottom dish with a density of 1 × 10^5^ cells per dish and incubated in an incubator with 5% CO_2_ for 24 h. Flavone derivatives (1000 μL, 10 μM) were added to the dish and incubated at 37 °C for 30 min, the medium was later removed, and the cells were washed three times with phosphate buffered saline (PBS) buffer. Then the cell imaging of these compounds were observed on a FV3000 laser scanning confocal microscope (Zeiss) using an oil immersion lens (60× magnification, NA 1.4) within a bandwidth of 460−560 nm. A549 cells were cultured for 24 h with the same way the above mentioned then flavone derivatives (10 μM) and 200 nM Mito Tracker Deep Red (a commercially available mitochondrial dye) were added to the dish and incubated for 30 min for colocalization experiments. Thirty min later, the solutions were removed and cells were washed with PBS three times. Last, cell imaging of these flavone derivatives were obtained on a confocal laser scanning microscope.

## 4. Conclusions

In summary, a novel AIEE system involving flavone-based derivatives was developed based on our previous study of AIEE molecules. They showed obvious AIEE characteristics, which were confirmed by their optical spectra, fluorescence photographs, fluorescence quantum yields (Ф_F_) and fluorescence lifetime. These compounds exhibit various fluorescence emissions ranging from purple, blue, cyan to green as the substituent groups change. These different colors offer great possibilities for their application in other aspects. Moreover, these AIEE compounds have excellent specificity for mitochondrial targeting and morphological change tracking, and they display low cytotoxicity at the experimental concentration range, which indicates their potential application prospects in mitochondrial imaging. Because of these excellent properties, further studies on the other applications such as their use as multifunctional probes and in vivo imaging are easy to carry out. Further efforts to discover more multipurpose AIEE fluorophores and their applications in living cell imaging and in vivo imaging are in progress.

## Figures and Tables

**Table 1:** Quantum yields (Ф_F_) and lifetimes (ns) of **5a**, **5b**, **5c**, **5d**, **5e** and **5f** in different CH_3_OH/H_2_O (*v*/*v*) mixtures (2.09 × 10^−5^ M).

Compound	Solvents	Quantum Yields (Ф_F_)	Time/ns
	CH_3_OH	0.16	3.15
**5a**	CH_3_OH/H_2_O (3:7)	0.23	3.22
	CH_3_OH/H_2_O (1:9)	0.19	3.19
	CH_3_OH	0.03	2.15
**5b**	CH_3_OH/H_2_O (5:5)	0.09	2.32
	CH_3_OH/H_2_O (1:9)	0.15	3.10
	CH_3_OH	0.14	2.97
**5c**	CH_3_OH/H_2_O (5:5)	0.22	3.29
	CH_3_OH/H_2_O (1:9)	0.28	3.34
	CH_3_OH	0.05	2.18
**5d**	CH_3_OH/H_2_O (5:5)	0.10	2.68
	CH_3_OH/H_2_O (1:9)	0.02	1.67
	CH_3_OH	0.20	3.26
**5e**	CH_3_OH/H_2_O (5:5)	0.27	3.31
	CH_3_OH/H_2_O (1:9)	0.32	3.40
	CH_3_OH	0.13	2.83
**5f**	CH_3_OH/H_2_O (4:6)	0.18	3.16
	CH_3_OH/H_2_O (1:9)	0.12	2.79

## Supplementary Materials

The following are available online. Figure S1: title PL spectra of **5g, 5h, 5i, 5j** in CH_3_OH/H_2_O mixtures, Figure S2: PL spectra of **5a**, **5b**, **5c**, **5d**, **5e** and **5f** in CH_3_OH/ethylene glycol mixtures, Figure S3: Time-dependent fluorescence spectra of **5a**, **5b**, **5c**, **5d**, **5e** and **5f** in CH_3_OH/H_2_O mixtures, Figure S4: Pearson’s correlation coefficient of **5a**, **5b**, **5c**, **5d**, **5e** and **5f** in A549 cells, Figure S5: ^1^H, ^13^C-NMR spectra and ESI-MS analysis for compounds **5a**–**5j**.

## References

[1] Li X., Jiang M., Lam J.W., Tang B., Qu Y.J. (2017). Mitochondrial Imaging with Combined Fluorescence and Stimulated Raman Scattering Microscopy Using a Probe of the Aggregation-Induced Emission Characteristic. J. Am. Chem. Soc..

[2] Sreedharan S., Gill M.R., Garcia E., Saeed H.K., Robinson D., Byrne A., Cadby A., Keyes T.E., Smythe C., Pellett P. (2017). Multimodal Super-Resolution Optical Microscopy Using a Transition-Metal-Based Probe Provides Unprecedented Capabilities for Imaging Both Nuclear Chromatin and Mitochondria. J. Am. Chem. Soc..

[3] Xu S., Wu W., Cai X., Zhang C., Yuan Y., Liang J., Feng G., Manghnani P., Liu B. (2017). Highly Efficient Photosensitizers with Aggregation-Induced Emission Characteristics Obtained through Precise Molecular Design. Chem. Commun..

[4] Li K., Ding D., Huo D., Pu K., Thao N.N., Hu Y., Li Z., Liu B. (2012). Conjugated Polymer Based Nanoparticles as Dual-Modal Probes for Targeted In Vivo Fluorescence and Magnetic Resonance Imaging. Adv. Funct. Mater..

[5] Zhang C., Feng G., Xu S., Zhu Z., Lu X., Wu J., Liu B. (2016). Structure-Dependent cis/trans Isomerization of Tetraphenylethene Derivatives: Consequences for Aggregation-Induced Emission. Angew. Chem. Int. Ed..

[6] Luo J., Xi Z., Lam J.W., Cheng L., Tang B., Chen H., Qiu C., Kwok H.S., Zhan X., Liu Y. (2001). Aggregation-Induced Emission of 1-methyl-1,2,3,4,5-pentaphenylsilole. Chem. Commun..

[7] Nishiuchi T., Tanaka K., Kuwatani Y., Sung J., Nishinaga T., Kim D., Iyoda M. (2013). Solvent-Induced Crystalline-State Emission and Multichromism of a Bent π-Surface System Composed of Dibenzocyclooctatetraene Units. Chem. Eur. J..

[8] Zhao Z., Lam J.W., Chan C.Y., Chen S., Liu J., Lu P., Rodriguez M., Maldonado J.L., Ramos-Ortiz G., Sung H.H. (2011). Stereoselective synthesis, efficient light emission, and high bipolar charge mobility of chiasmatic luminogens. Adv. Mater..

[9] Lu H., Xu B., Dong Y., Chen F., Li Y., Li Z., He J., Li H., Tian W. (2010). Novel fluorescent pH sensors and a biological probe based on anthracene derivatives with aggregation-induced emission characteristics. Langmuir.

[10] Mei J., Hong Y., Lam J.W., Qin A., Tang Y., Tang B. (2014). Aggregation-Induced Emission: The Whole Is More Brilliant than the Parts. Adv. Mater..

[11] Leung N.L., Xie N., Yuan W., Liu Y., Wu Q., Peng Q., Miao Q., Lam J.W., Tang B. (2014). Restriction of Intramolecular Motions: The General Mechanism behind Aggregation-Induced Emission. Chem. Eur. J..

[12] Zhao E., Lam J.W., Hong Y., Liu J., Peng Q., Hao J., Sung H.H., Williams I.D., Tang B. (2013). How Do Substituents Affect Silole Emission?. J. Mater. Chem. C.

[13] Jiang G., Zeng G., Zhu W., Li Y., Dong X., Zhang G., Fan X., Wang J., Wu Y., Tang B. (2017). A selective and light-up fluorescent probe for β-galactosidase activity detection and imaging in living cells based on an AIE tetraphenylethylene derivative. Chem. Commun..

[14] Chowdhury A., Howlader P., Mukherjee P.S. (2016). Aggregation-Induced Emission of Platinum(II) Metallacycles and Their Ability to Detect Nitroaromatics. Chem. Eur. J..

[15] Zhang C., Jin S., Yang K., Xue X., Li Z., Jiang Y., Chen W., Dai L., Zou G., Liang X. (2014). Cell membrane tracker based on restriction of intramolecular rotation. ACS Appl. Mater. Interfaces.

[16] Jiang G., Wang J., Yang Y., Zhang G., Liu Y., Lin H., Zhang G., Li Y., Fan X. (2016). Fluorescent turn-on sensing of bacterial lipopolysaccharide in artificial urine sample with sensitivity down to nanomolar by tetraphenylethylene based aggregation induced emission molecule. Biosens. Bioelectron..

[17] Lei Y., Liu L., Tang X., Yang D., Yang X., He F. (2018). Sanguinarine and chelerythrine: Two natural products for mitochondria-imaging with aggregation-induced emission enhancement and pH-sensitive characteristics. RSC Adv..

[18] Thompson R.B. (2005). Fluorescence Sensors and Biosensors.

[19] Zhang L., Liu W., Huang X., Zhang G., Wang X., Wang Z., Zhang D., Jiang X. (2015). Old is new again: A chemical probe for targeting mitochondria and monitoring mitochondrial membrane potential in cells. Analyst.

[20] Hu R., Leung N.L., Tang B. (2014). AIE Macromolecules: Syntheses, Structures and Functionalities. Chem. Soc. Rev..

[21] Masahito H., Yoshie H. (2007). Direct observation of the reversible unwinding of a single DNA molecule caused by the intercalation of ethidium bromide. Nucleic Acids Res..

[22] Björn O., Patrick M., Per A., Michael F., Fabrizio G., Gunnar G., Constanze H., Maurice D.L., Par M., Giovanni M. (2014). Impact of Stereospecific Intramolecular Hydrogen Bonding on Cell Permeability and Physicochemical Properties. J. Med. Chem..

[23] Tang X., Zhang J., Liu L., Yang D., Wang H., He F. (2017). Synthesis of 13-substituted derivatives of berberine:Aggregation-induced emission enhancement and pH sensitive property. J. Photochem. Photobiol. A.

[24] Jiang W., Luo T., Li S., Zhou Y., Shen X., He F. (2016). Quercetin Protects against Okadaic Acid-Induced Injury via MAPK and PI3K/Akt/GSK3β Signaling Pathways in HT22 Hippocampal Neurons. PLoS ONE.

[25] Li J., Nahyun K., Yerin J., Songyi L., Gyoungmi K., Juyoung Y. (2018). Aggregation-Induced Fluorescence Probe for Monitoring Membrane Potential Changes in Mitochondria. ACS Appl. Mater. Interfaces.

[26] Ding D., Goh C., Feng G., Zhao Z., Liu J., Liu R., Tomczak N., Geng J., Tang B., Ng L.G. (2013). Ultrabright Organic Dots with Aggregation-Induced Emission Characteristics for Real-Time Two-Photon Intravital Vasculature Imaging. Adv. Mater..

[27] Ding D., Li K., Liu B., Tang B. (2013). Bioprobes Based on AIE Fluorogens. Acc. Chem. Res..

[28] Hong Y., Lam J.W., Tang B. (2011). Aggregation-Induced Emission. Chem. Soc. Rev..

[29] Yuan Y., Kwok R.T., Tang B., Liu B. (2014). Targeted Theranostic Platinum (IV) Prodrug with a Built-in Aggregation-Induced Emission Light-Up Apoptosis Sensor for Noninvasive Early Evaluation of its Therapeutic Responses in Situ. J. Am. Chem. Soc..

[30] Hu F., Huang Y., Zhang G., Zhao R., Yang H., Zhang D. (2014). Targeted Bioimaging and Photodynamic Therapy of Cancer Cells with an Activatable Red Fluorescent Bioprobe. Anal. Chem..

[31] Zhang S., Qin A., Sun J., Tang B. (2011). Mechanism study of aggregation-induced emission. Prog. Chem..

[32] Ghodbane A., D’Alterio S., Saffon N., McClenaghan N.D., Scarpantonio L., Jolinat P., Fery-Forgues S. (2012). Facile access to highly fluorescent nanofibers and microcrystals via reprecipitation of 2-phenyl-benzoxazole derivatives. Langmuir.

[33] Dong J., Solntsev K.M., Tolbert L.M. (2009). Activation and tuning of green fluorescent protein chromophore emission by alkyl substituent-mediated crystal packing. J. Am. Chem. Soc..

[34] Li K., Zhu Z., Cai P., Liu R., Tomczak N., Ding D., Liu J., Qin W., Zhao Z., Hu Y. (2013). Organic Dots with Aggregation-Induced Emission (AIE dots) Characteristics for Dual-Color Cell Tracing. Chem. Mater..

[35] Gu X., Yao J., Zhang G., Zhang C., Yan Y., Zhao Y., Zhang D. (2013). New Electron-Donor/Acceptor-Substituted Tetraphenylethylenes: Aggregation-Induced Emission with Tunable Emission Color and Optical-Waveguide Behavior. Chem. Asian J..

[36] Zhao N., Chen S., Hong Y., Tang B. (2015). A red emitting mitochondria-targeted AIE probe as an indicator for membrane potential and mouse sperm activity. Chem. Commun..

[37] Chris W.T., Hong Y., Chen S., Zhao E., Jacky W.Y., Tang B. (2013). A Photostable AIE Luminogen for Specific Mitochondrial Imaging and Tracking. J. Am. Chem. Soc..

[38] Lim M.L., Minamikawa T., Nagley P. (2001). The protonophore CCCP induces mitochondrial permeability transition without cytochrome c release in human osteosarcoma cells. FEBS Lett..

[39] Yao N., Song A., Wang X., Seth D., Kit S.L. (2007). Synthesis of Flavonoid Analogues as Scaffolds for Natural Product-Based Combinatorial Libraries. J. Comb. Chem..

[40] Cotelle N., Bernier J.L., Henichart J.P., Catteau J.P., Gaydou E., Wallet J.C. (1992). Scavenger and antioxidant properties of ten synthetic flavones. Free Radic. Biol. Med..

[41] Chen J., Wu W. (2013). Fluorescent Polymeric Micelles with Aggregation-Induced Emission Properties for Monitoring the Encapsulation of Doxorubicin. Macromol. Biosci..

[42] Xue X., Zhao Y., Dai L., Zhang X., Hao X., Zhang C., Huo S., Liu J., Liu C., Kumar A. (2014). Spatiotemporal Drug Release Visualized through a Drug Delivery System with Tunable Aggregation-Induced Emission. Adv. Mater..

[43] Li Z., Dong Y., Mi B., Tang Y., Häussler M., Tong H., Dong Y., Lam J.W., Ren Y., Sung H.H. (2005). Structure Control of the Photoluminescence of Silole Regioisomers and Their Utility as Sensitive Regiodiscriminating Chemosensors and Efficient Electroluminescent Materials. J. Phys. Chem. B.

