# Supplementary material for: Rational Design for Multicolor Flavone-Based Fluorophores with Aggregation-Induced Emission Enhancement Characteristics and Applications in Mitochondria-Imaging
Source: Molecules. 2018 Sep 7;23(9):2290. doi: 10.3390/molecules23092290 (PMC6225312; doi:10.3390/molecules23092290)
Supplement: Supplementary file 1 [file molecules-23-02290-s001.pdf]

Supplementary Information

Rational design for multicolor flavone-based fluorophores with Aggregation-induced emission enhancement characteristics and application for mitochondria-imaging

Liyan Liu[1], Yaohui Lei[1], Jianhui Zhang[2], Na Li[1], FanZhang[1],Huaqiao Wang[3] andFeng He[1,*]

[1]School of Pharmaceutical Science, Sun Yat-sen University, Guangzhou 510006, China.

[2] School of Chemistry, Sun Yat-sen University, Guangzhou 510275, China.

[3] Department of Anatomy and Neurobiology, Zhongshan School of Medicine, Sun Yat-sen University, Guangzhou 510080, China.

* Correspondence: hefeng@mail.sysu.edu.cn; Tel.: +86-203-994-3036

Table of Contents

PL spectra of **5g, 5h, 5i, 5j** in CH_3OH/H_2O mixtures ………..……......................Figure S1

PL spectra of **5a, 5b, 5c, 5d, 5e, 5f**in CH_3OH/ethylene glycol mixtures ……....Figure S2

Time-dependent fluorescence spectra of **5a**, **5b**, **5c**, **5d**, **5e** and **5f** in CH_3OH/H_2O mixtures ……..Figure S3

Pearson's correlation coefficient of **5a, 5b, 5c, 5d, 5e, 5f** in A549 cells…..............Figure S4

^1H, ^{13}C NMR spectra and ESI-MS analysis for compound **5a**………...................Figure S5

^1H, ^{13}C NMR spectra and ESI-MS analysis for compound **5b**………...……........Figure S6

^1H, ^{13}C NMR spectra and ESI-MS analysis for compound **5c**………...……..........Figure S7

^1H, ^{13}C NMR spectra and ESI-MS analysis for compound **5d**………...................Figure S8

^1H, ^{13}C NMR spectra and ESI-MS analysis for compound **5e**………...…............Figure S9

^1H, ^{13}C NMR spectra and ESI-MS analysis for compound **5f**………...….............Figure S10

^1H, ^{13}C NMR spectra and ESI-MS analysis for compound **5g**………...…...........Figure S11

^1H, ^{13}C NMR spectra and ESI-MS analysis for compound **5h**………...…...........Figure S12

^1H, ^{13}C NMR spectra and ESI-MS analysis for compound **5i**………...…............Figure S13

^1H, ^{13}C NMR spectra and ESI-MS analysis for compound **5j**………...…............Figure S14

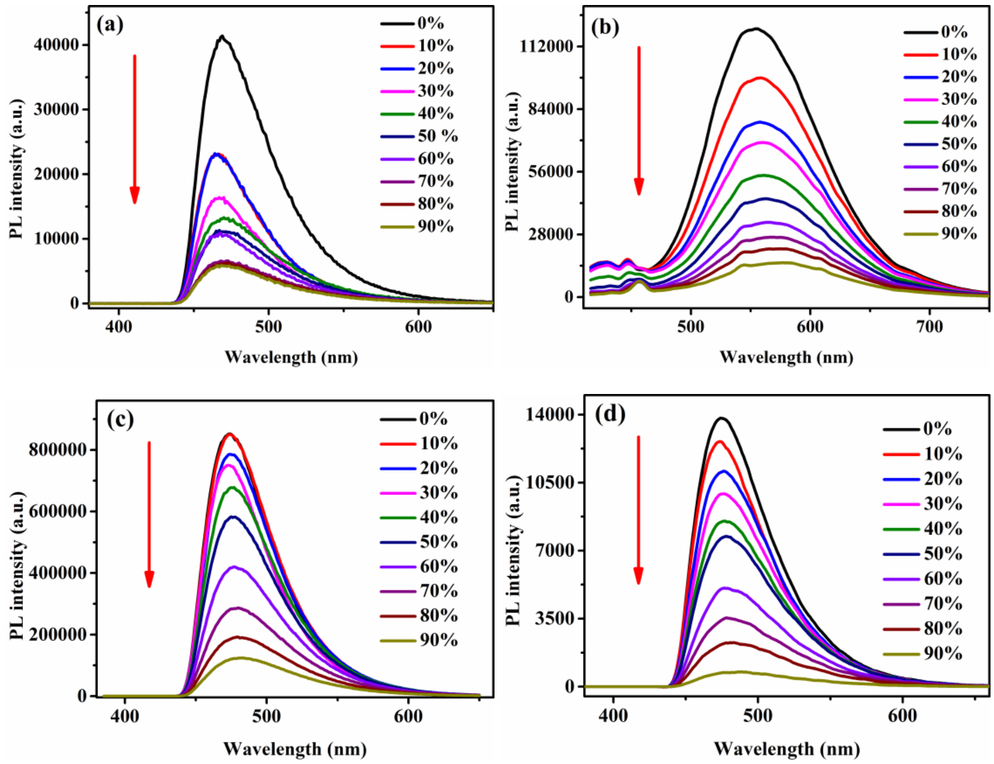

Figure S1. PL spectra of (a) **5g**, (b) **5h**, (c) **5i**, (d) **5j** (c = 2.09 × 10^{-5} M) in CH_3OH/H_2O mixtures with different water fractions (0-90 vol%).

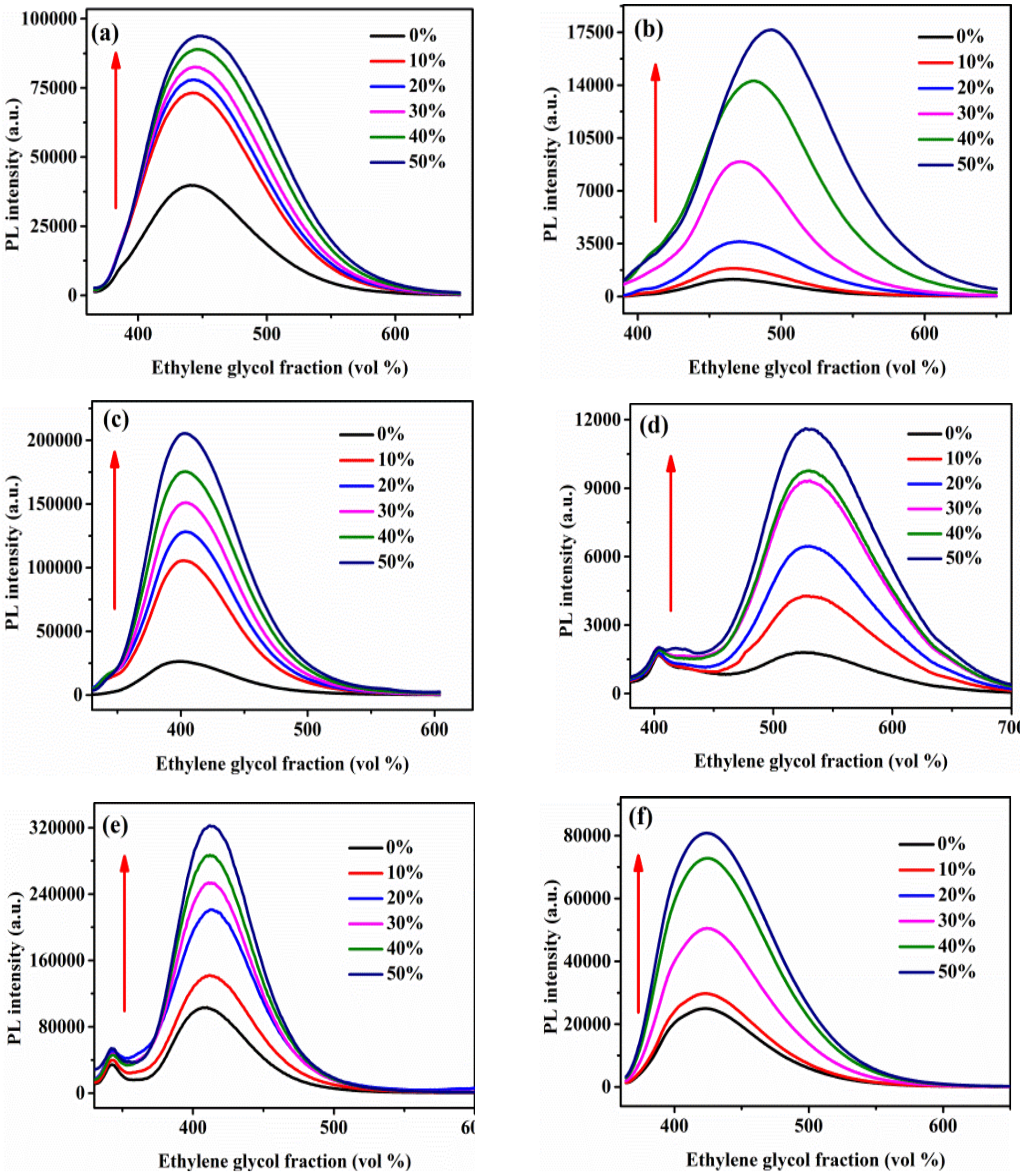

Figure S2. PL spectra of (a) **5a**, (b) **5b**, (c) **5c**, (d) **5d**, (e) **5e**, (f) **5f**. (c = 2.09×10^{-5} M) in CH_3OH/ ethylene glycol mixtures with different ethylene glycol fractions (0-50 vol%).

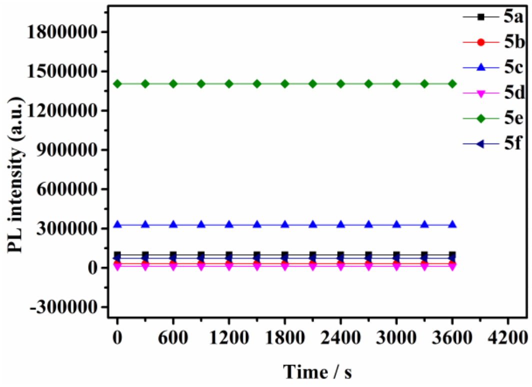

Figure S3. Time-dependent fluorescence spectra of **5a** (3: 7 v: v), **5b** (1: 9 v: v), **5c** (1: 9 v: v) , **5d** (5: 5 v: v), **5e** (1: 9 v: v) and **5f** (4: 6 v: v) in CH_3OH/H_2O (v: v) mixtures (c = 2.09 × 10^{-5} M).

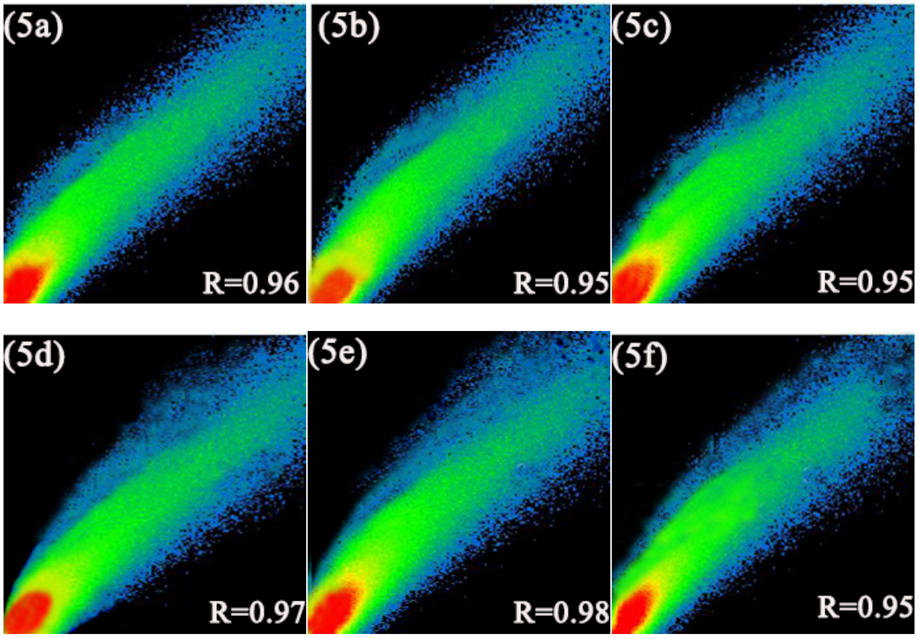

Figure S4.Pearson's correlation coefficient of **5a**, **5b**, **5c**, **5d**, **5e**, **5f** in A549 cells.

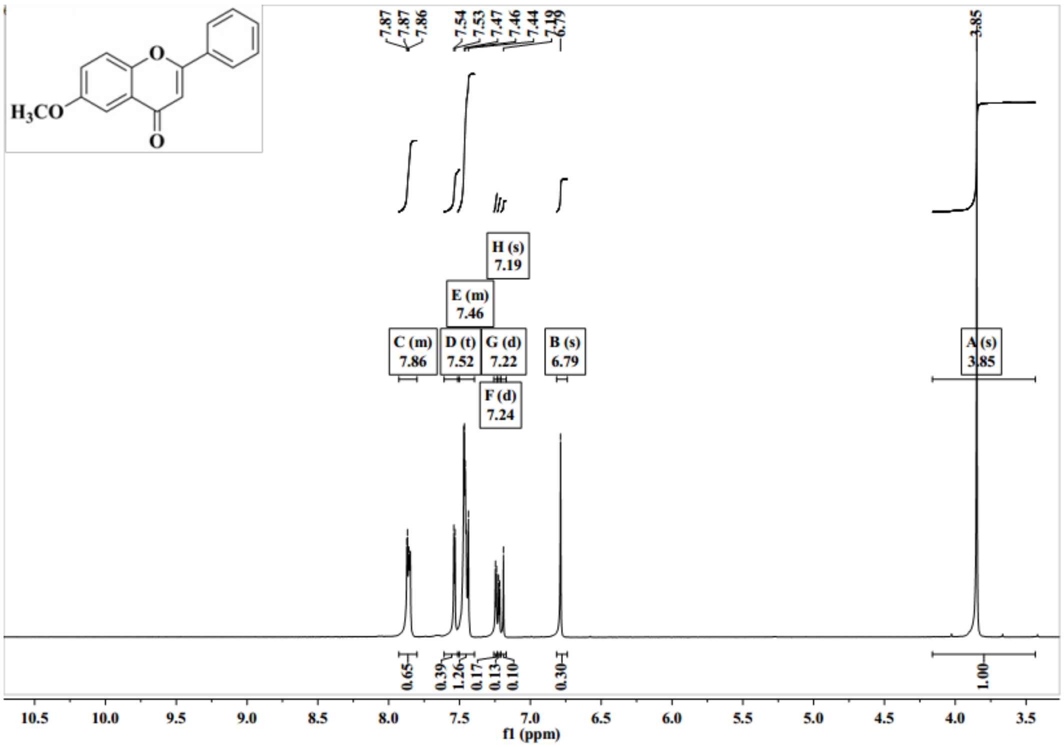
H3CO
7.87
7.87
7.86
7.54
7.53
7.47
7.46
7.44
7.19
6.79
3.85
H (s)
7.19
E (m)
7.46
C (m)
7.86
D (t)
7.52
G (d)
7.22
B (s)
6.79
A (s)
3.85
F (d)
7.24
0.65
0.39
1.26
0.17
0.13
0.10
0.30
1.00
10.5 10.0 9.5 9.0 8.5 8.0 7.5 7.0 6.5 6.0 5.5 5.0 4.5 4.0 3.5
f1 (ppm)

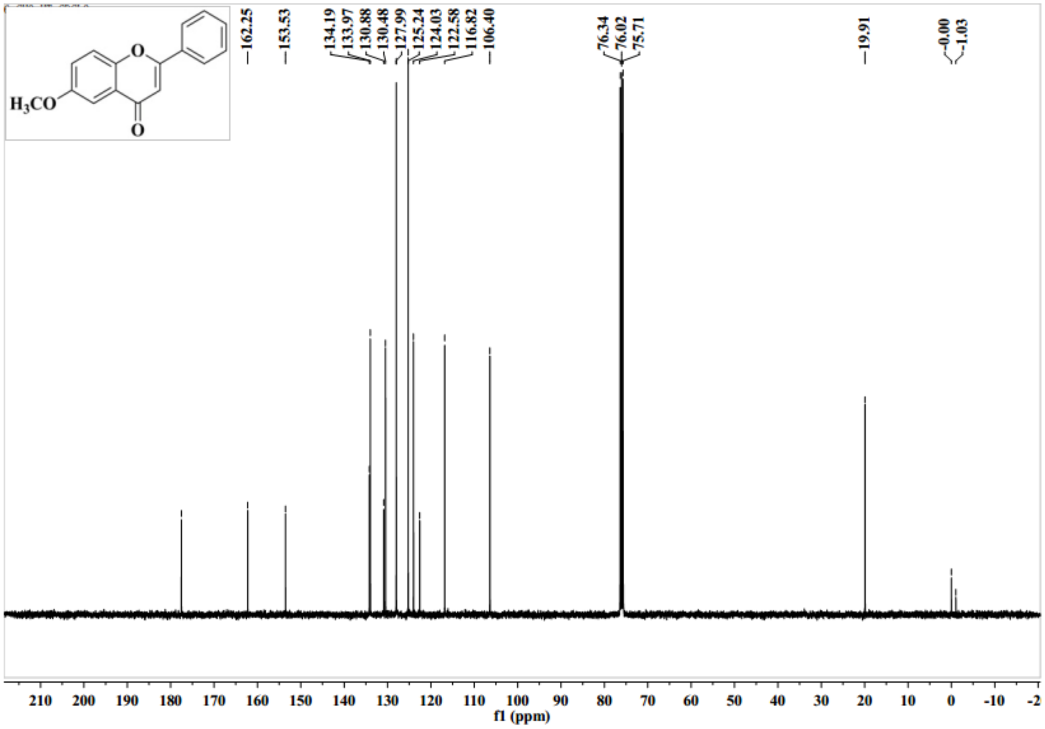
H3CO
162.25
153.53
134.19
133.97
130.88
130.48
127.99
125.24
124.03
122.58
116.82
106.40
76.34
76.02
75.71
19.91
-0.00
-1.03
210 200 190 180 170 160 150 140 130 120 110 100 90 80 70 60 50 40 30 20 10 0 -10 -2
f1 (ppm)

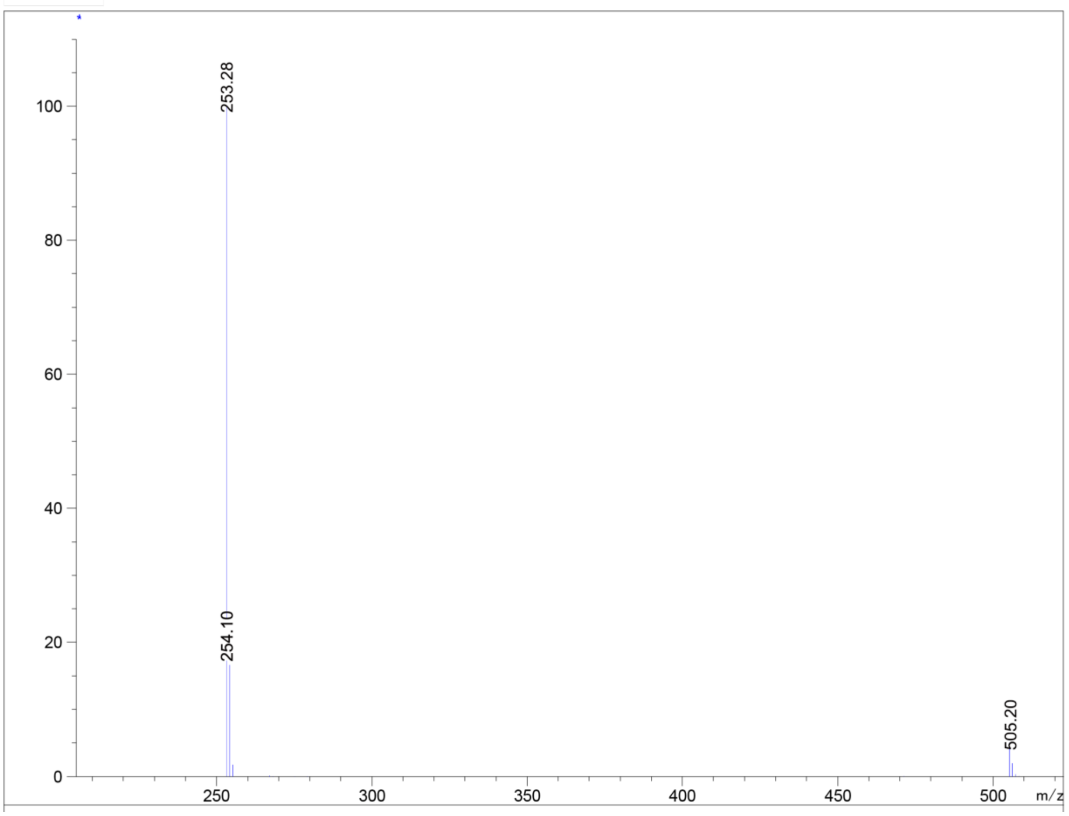

Figure S5.^{1}H, ^{13}C NMR spectra and ESI-MS analysis for compound **5a.**

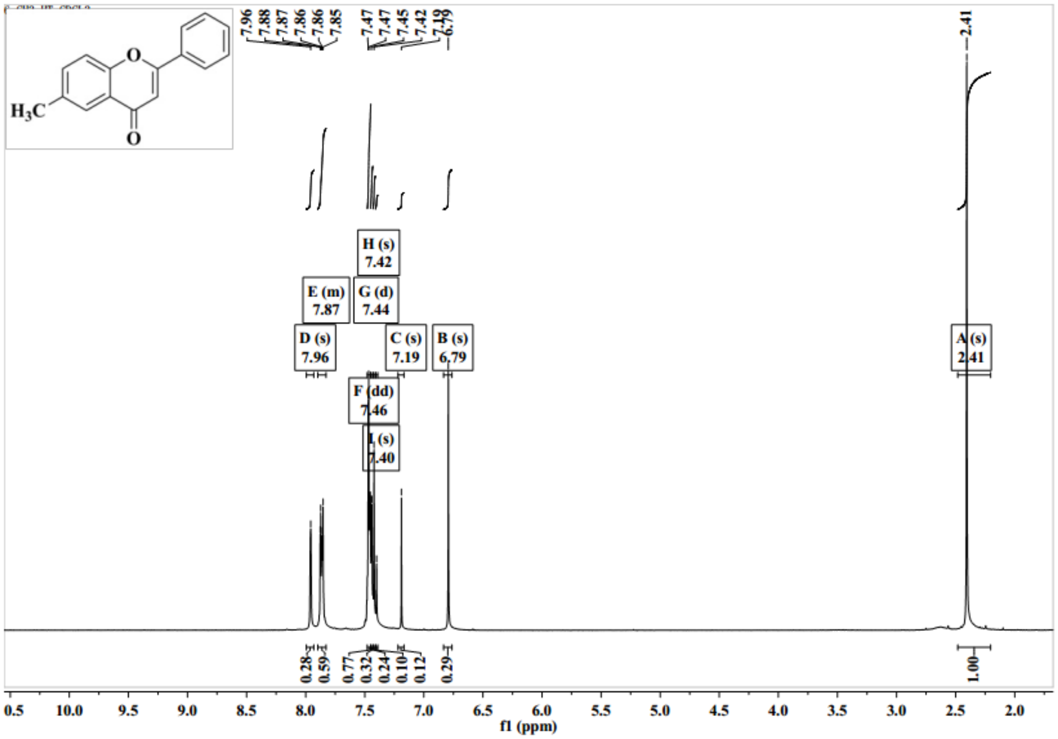
H$_3$C
O
O
7.96
7.88
7.87
7.86
7.86
7.85
7.47
7.47
7.45
7.42
7.19
6.79
2.41
H (s) 7.42
E (m) 7.87
G (d) 7.44
D (s) 7.96
C (s) 7.19
B (s) 6.79
A (s) 2.41
F (dd) 7.46
I (s) 7.40
0.28
0.59
0.77
0.32
0.24
0.10
0.12
0.29
1.00
f1 (ppm)

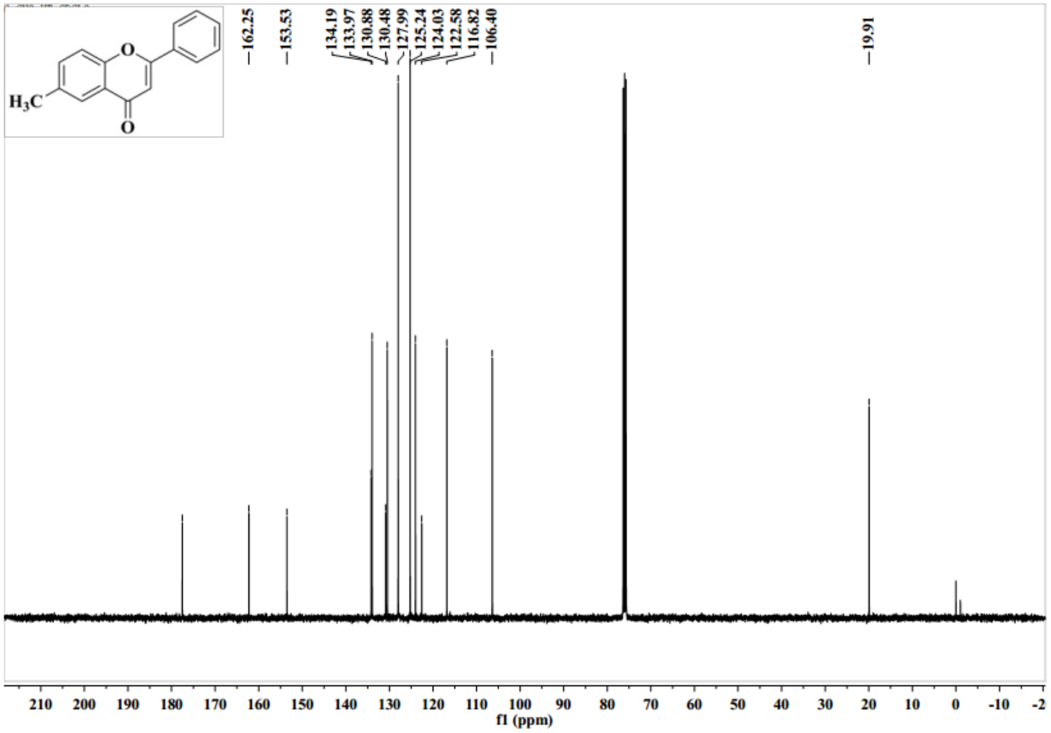
H$_3$C
O
O
162.25
153.53
134.19
133.97
130.88
130.48
127.99
125.24
124.03
122.58
116.82
106.40
19.91
f1 (ppm)

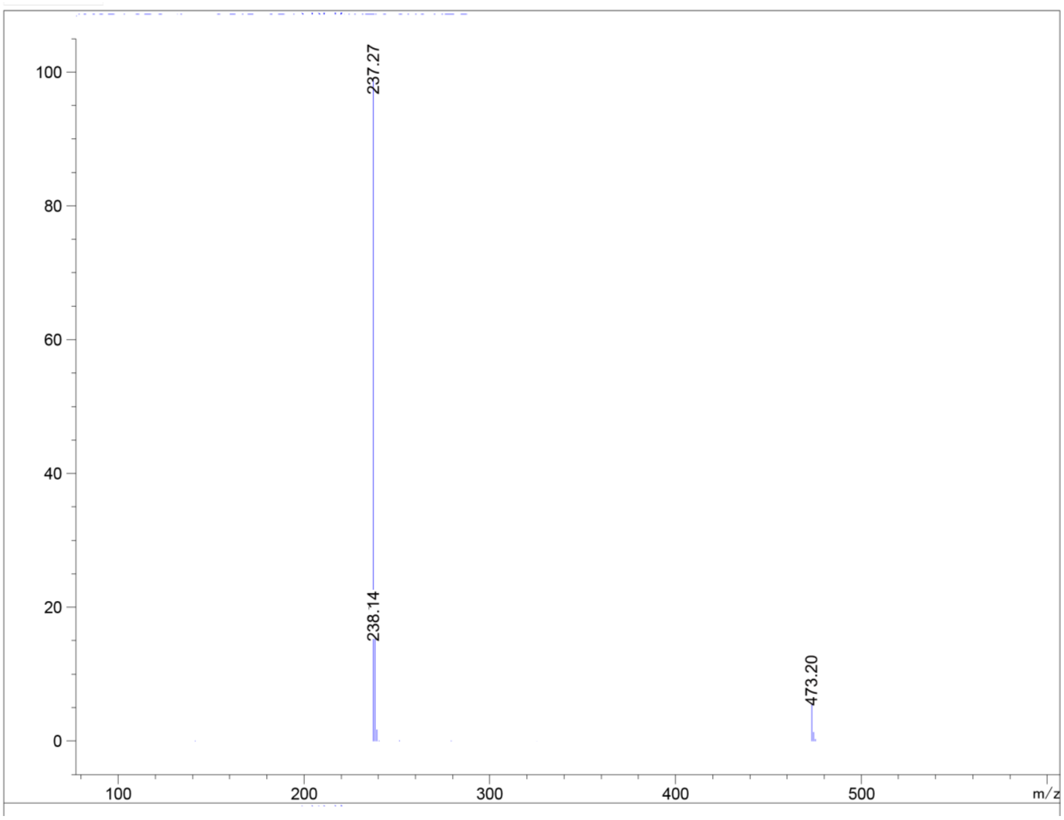

Figure S6.^{1}H, ^{13}C NMR spectra and ESI-MSanalysis for compound **5b.**

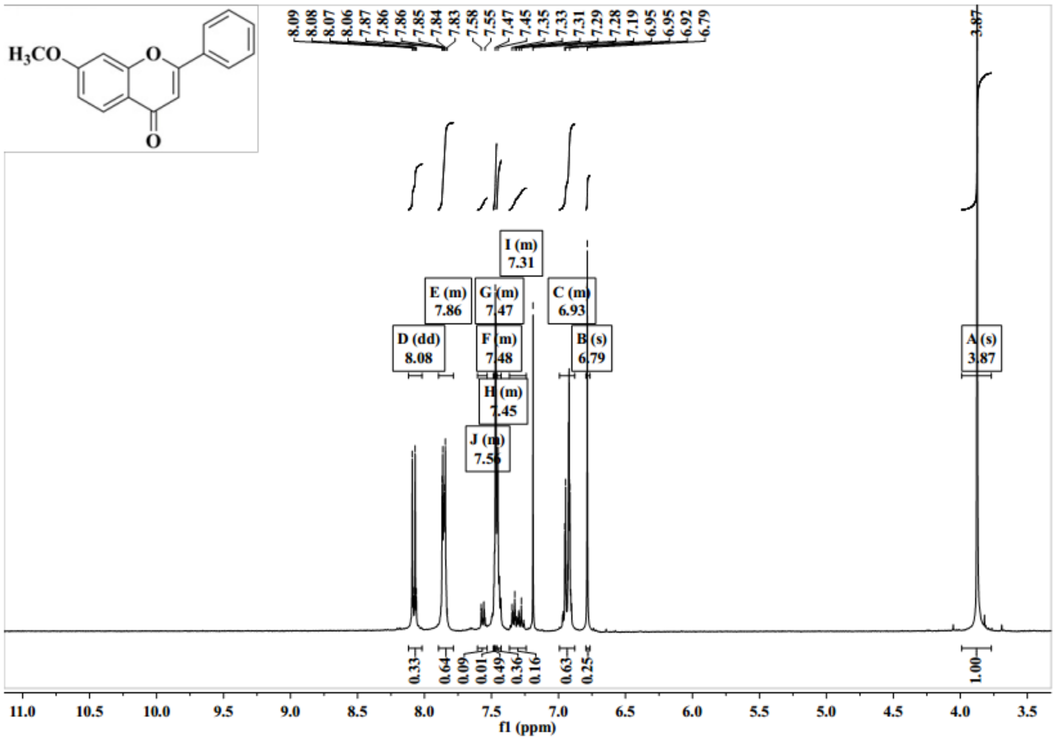
H3CO
O
8.09 8.08 8.07 8.06 7.87 7.86 7.86 7.85 7.84 7.83 7.58 7.55 7.47 7.45 7.35 7.33 7.31 7.29 7.28 7.19 6.95 6.95 6.92 6.79
3.87
I (m) 7.31
E (m) 7.86
G (m) 7.47
C (m) 6.93
D (dd) 8.08
F (m) 7.48
B (s) 6.79
A (s) 3.87
H (m) 7.45
J (m) 7.56
0.33 0.64 0.09 0.01 0.49 0.36 0.16 0.63 0.25
1.00
11.0 10.5 10.0 9.5 9.0 8.5 8.0 7.5 7.0 6.5 6.0 5.5 5.0 4.5 4.0 3.5
f1 (ppm)

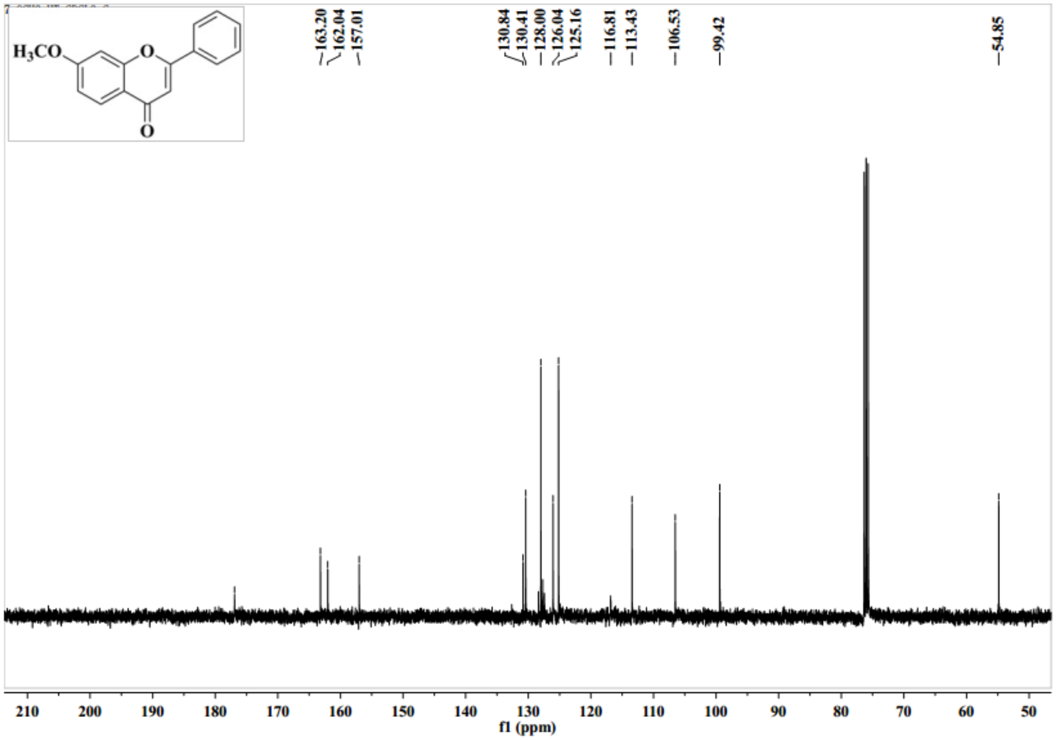
H3CO
O
163.20 162.04 157.01
130.84 130.41 128.00 126.04 125.16
116.81 113.43
106.53
99.42
54.85
210 200 190 180 170 160 150 140 130 120 110 100 90 80 70 60 50
f1 (ppm)

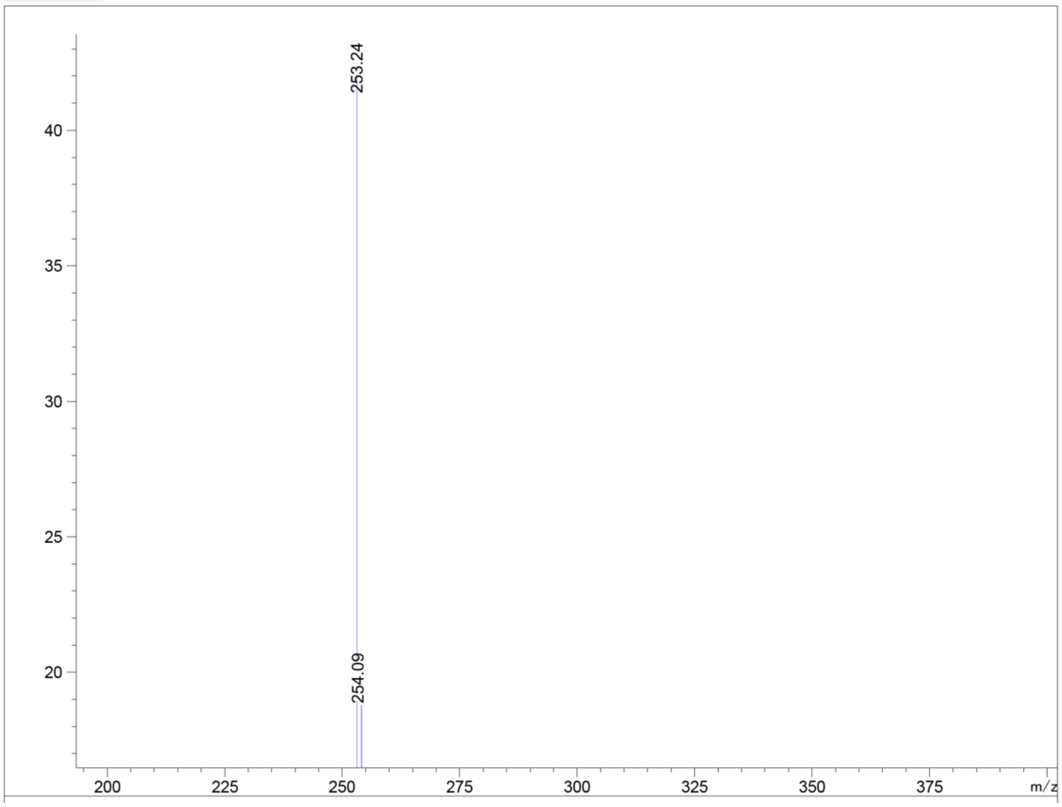

Figure S7.^{1}H, ^{13}C NMR spectra and ESI-MSanalysis for compound **5c.**

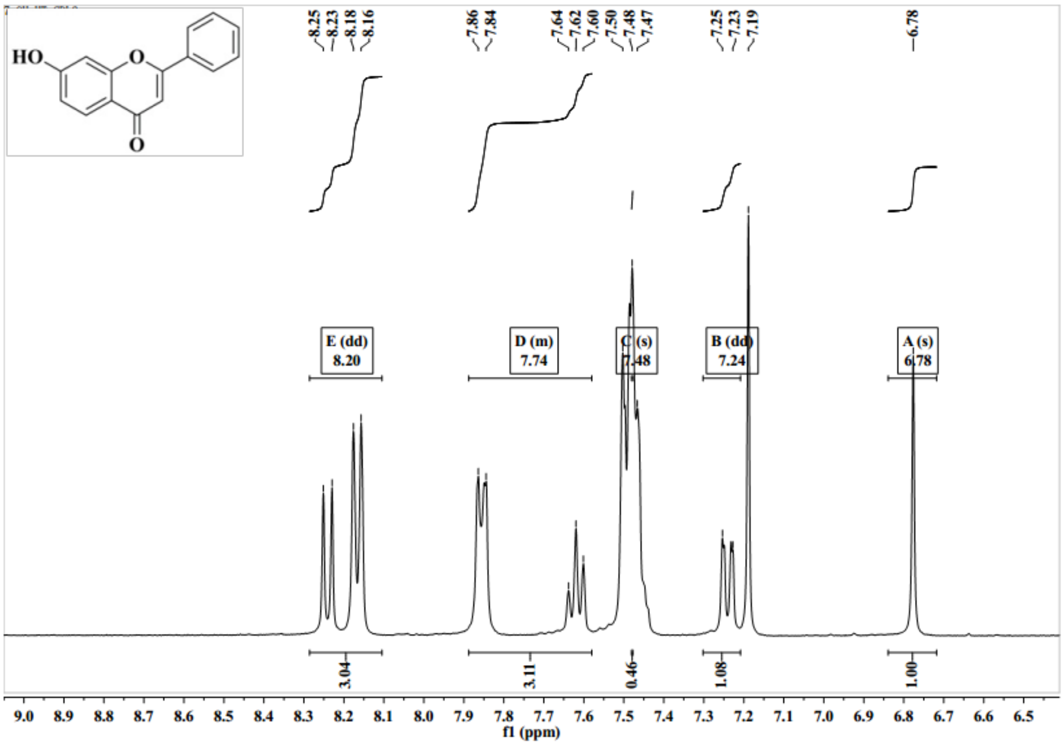
HO
O
O
8.25
8.23
8.18
8.16
7.86
7.84
7.64
7.62
7.60
7.50
7.48
7.47
7.25
7.23
7.19
6.78
E (dd)
8.20
D (m)
7.74
C (s)
7.48
B (dd)
7.24
A (s)
6.78
3.04
3.11
0.46
1.08
1.00
f1 (ppm)

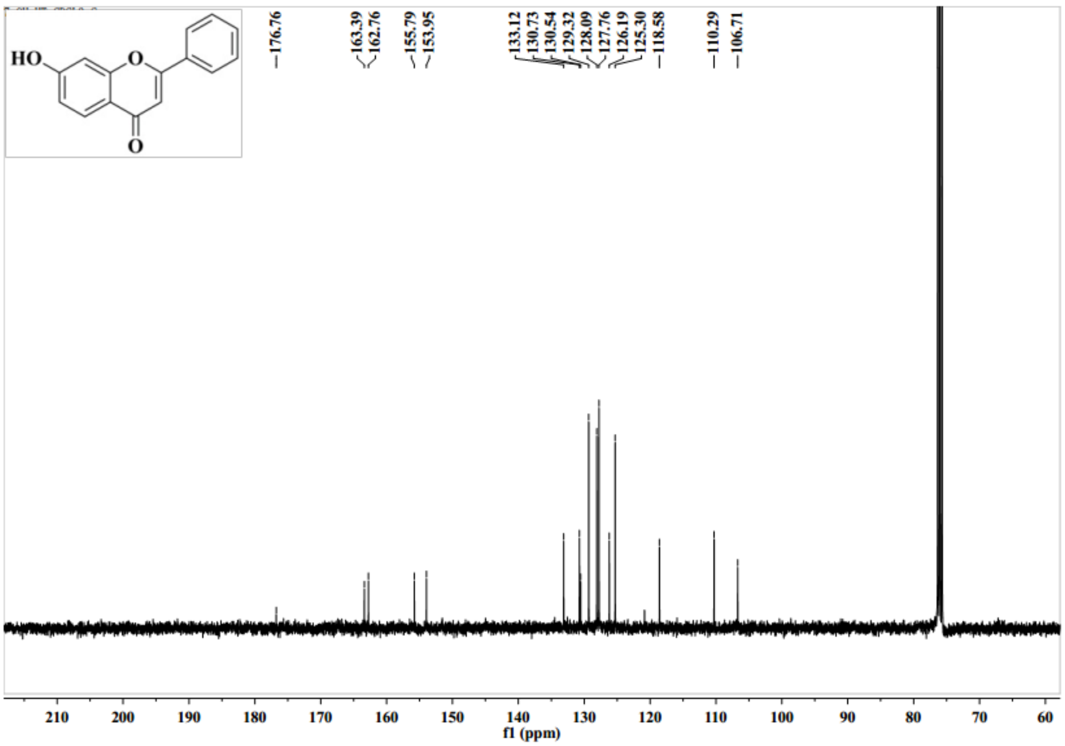
HO
O
O
176.76
163.39
162.76
155.79
153.95
133.12
130.73
130.54
129.32
128.09
127.76
126.19
125.30
118.58
110.29
106.71
f1 (ppm)

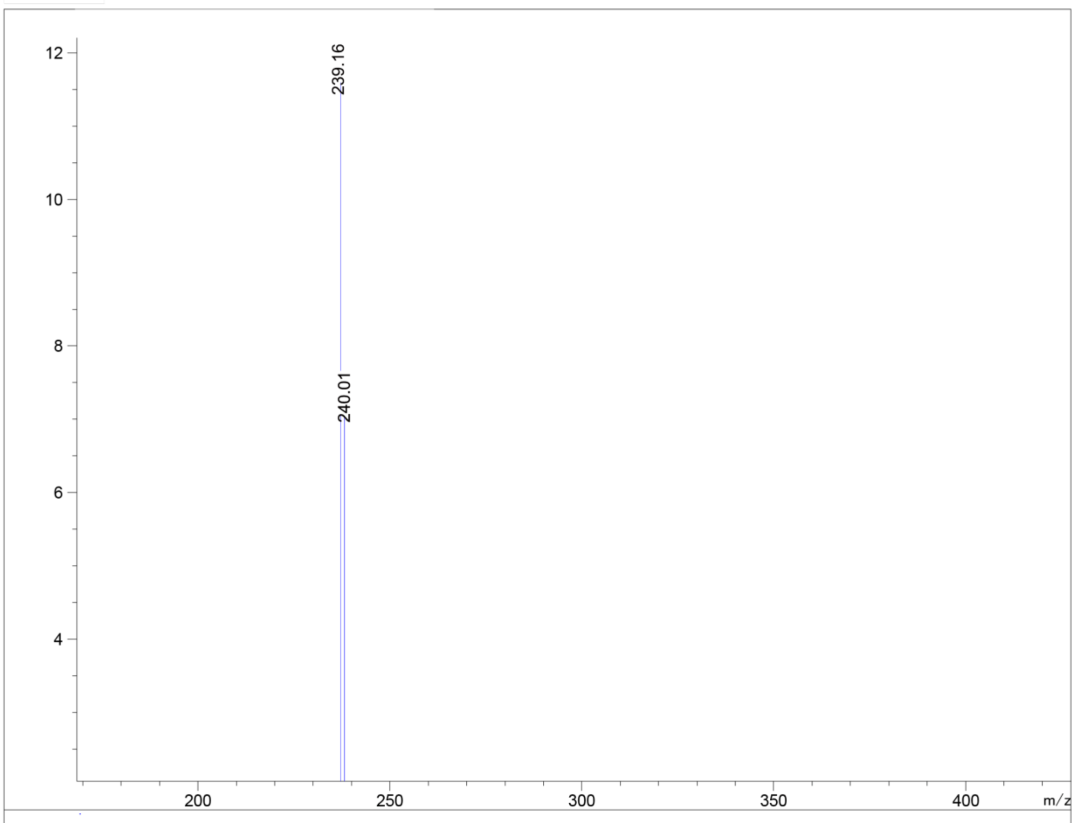

Figure S8.^{1}H, ^{13}C NMR spectra and ESI-MS analysis for compound **5d.**

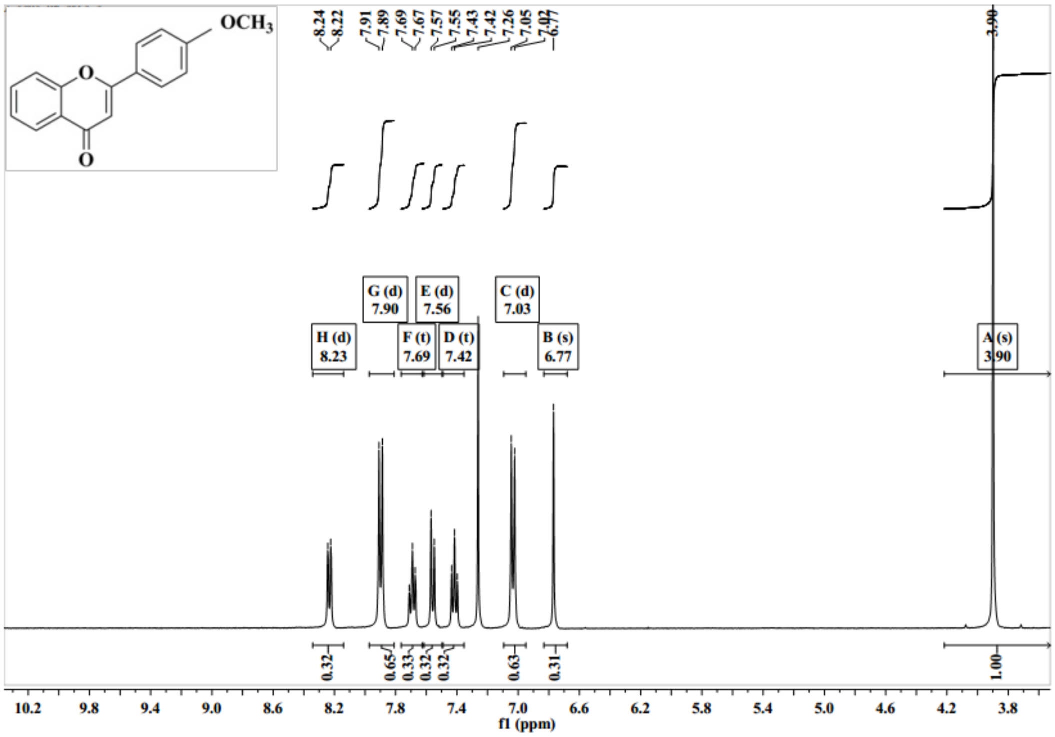
OCH3
O
O
8.24
8.22
7.91
7.89
7.69
7.67
7.57
7.55
7.43
7.42
7.26
7.05
7.02
6.77
3.90
H (d)
8.23
G (d)
7.90
F (t)
7.69
E (d)
7.56
D (t)
7.42
C (d)
7.03
B (s)
6.77
A (s)
3.90
0.32
0.65
0.33
0.32
0.32
0.63
0.31
1.00
f1 (ppm)

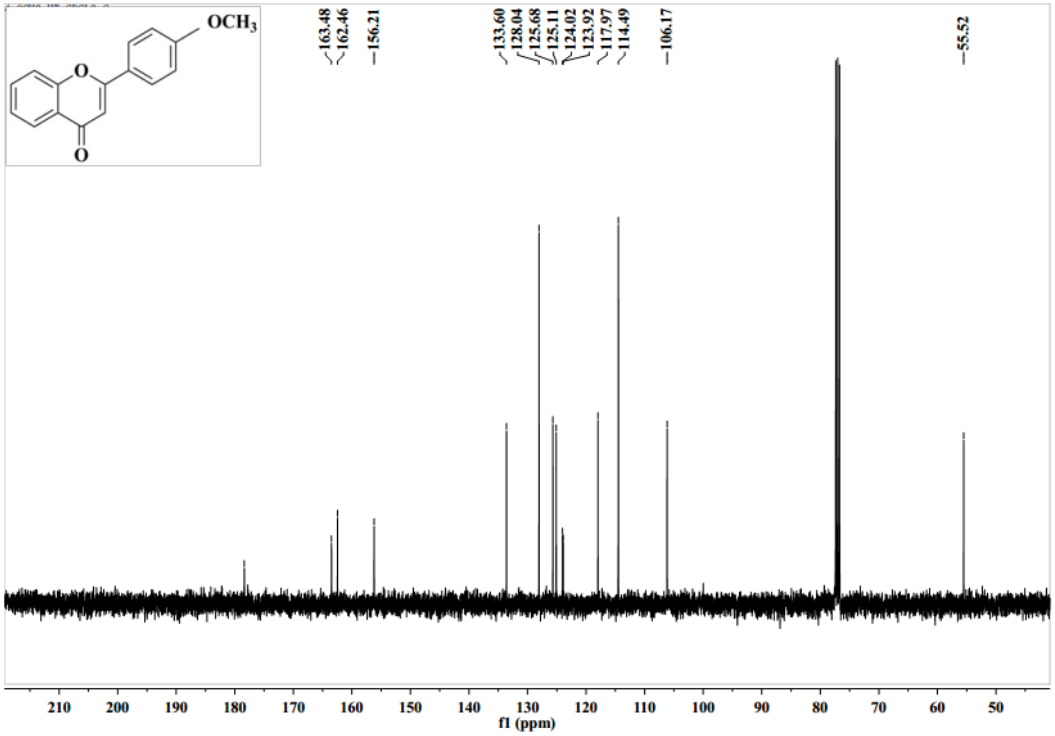
OCH3
O
O
163.48
162.46
156.21
133.60
128.04
125.68
125.11
124.02
123.92
117.97
114.49
106.17
55.52
f1 (ppm)

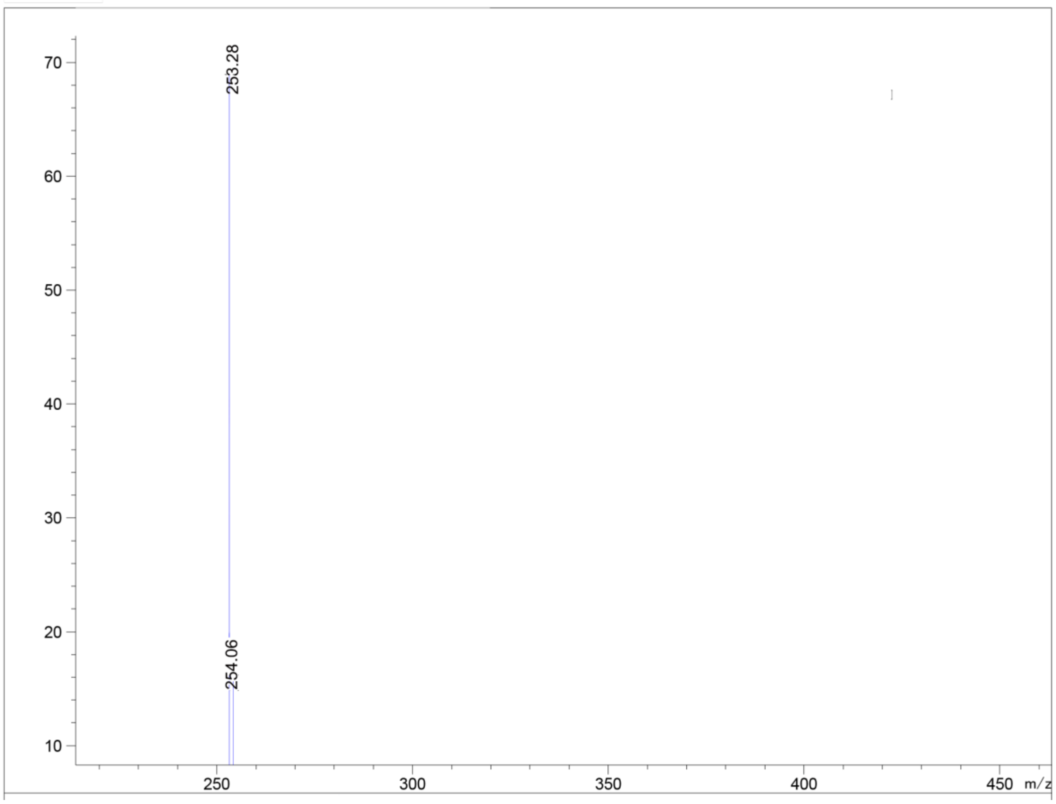

Figure S9. ^{1}H, ^{13}C NMR spectra and ESI-MS analysis for compound **5e.**

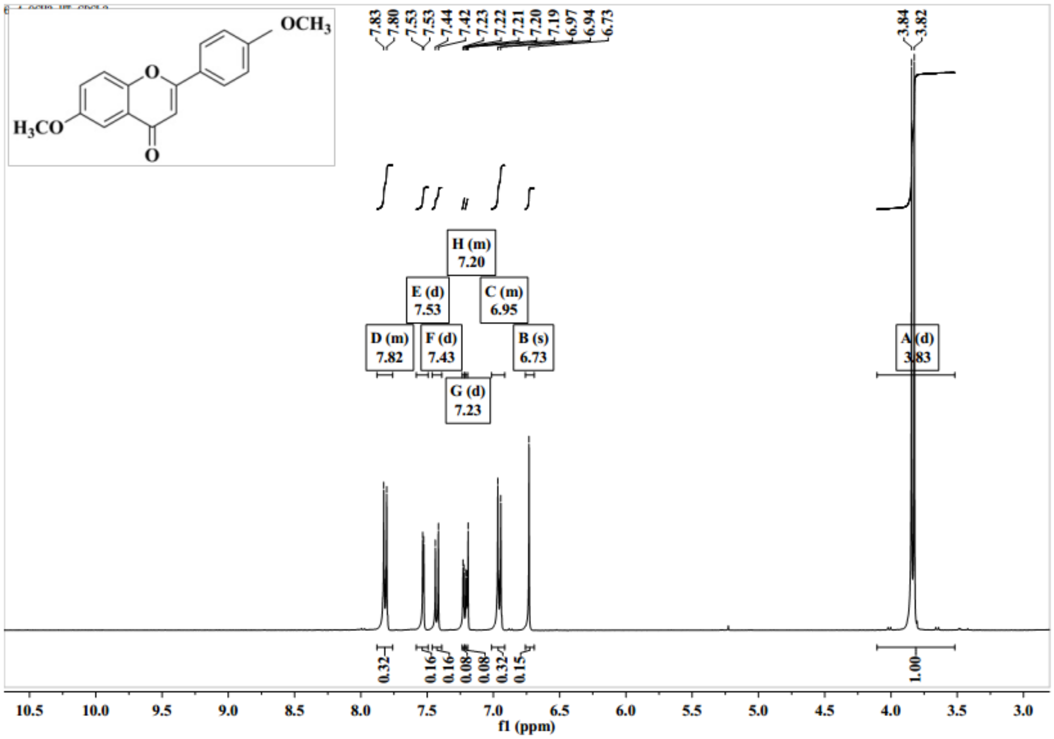
OCH3
H3CO
O
O
7.83
7.80
7.53
7.53
7.44
7.42
7.23
7.22
7.21
7.20
7.19
6.97
6.94
6.73
3.84
3.82
H (m)
7.20
E (d)
7.53
C (m)
6.95
D (m)
7.82
F (d)
7.43
B (s)
6.73
G (d)
7.23
A (d)
3.83
0.32
0.16
0.16
0.08
0.08
0.32
0.15
1.00
10.5
10.0
9.5
9.0
8.5
8.0
7.5
7.0
6.5
6.0
5.5
5.0
4.5
4.0
3.5
3.0
f1 (ppm)

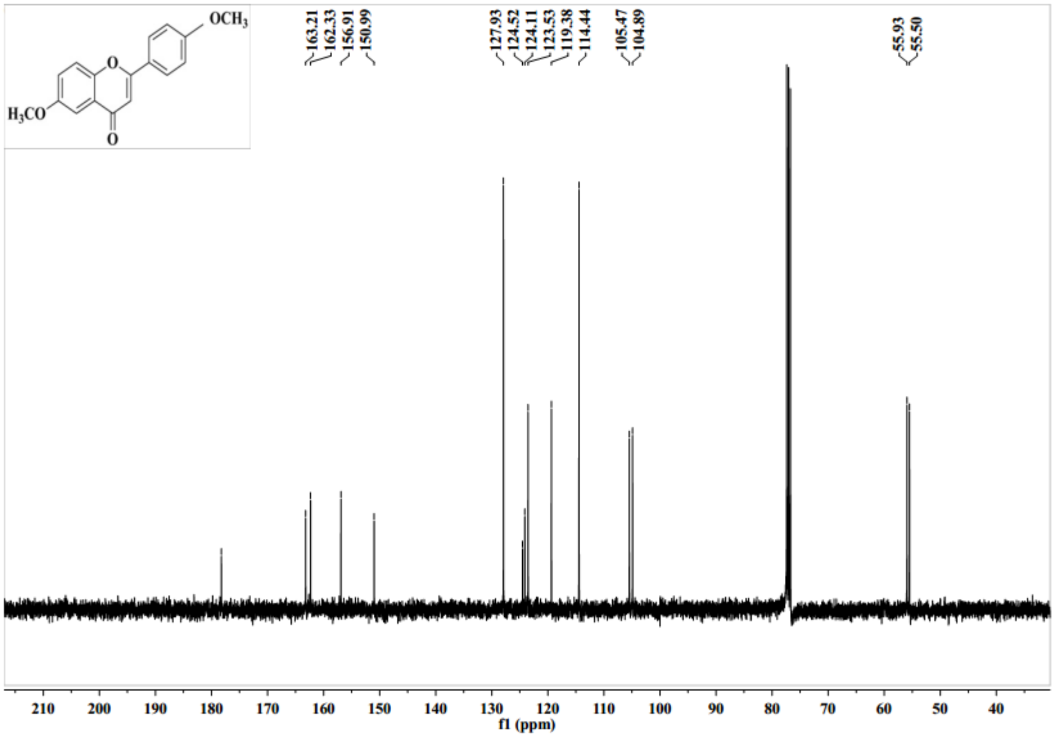
OCH3
H3CO
O
O
163.21
162.33
156.91
150.99
127.93
124.52
124.11
123.53
119.38
114.44
105.47
104.89
55.93
55.50
210
200
190
180
170
160
150
140
130
120
110
100
90
80
70
60
50
40
f1 (ppm)

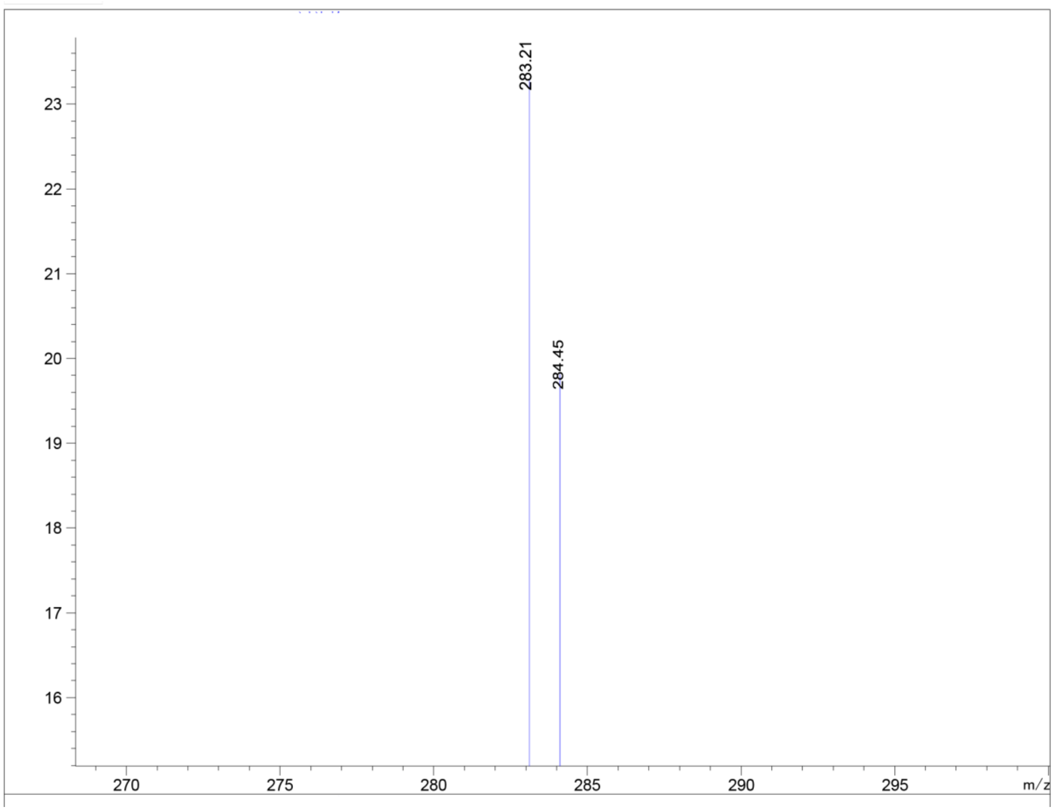

Figure S10.^{1}H, ^{13}C NMR spectra and ESI-MS analysis for compound **5f.**

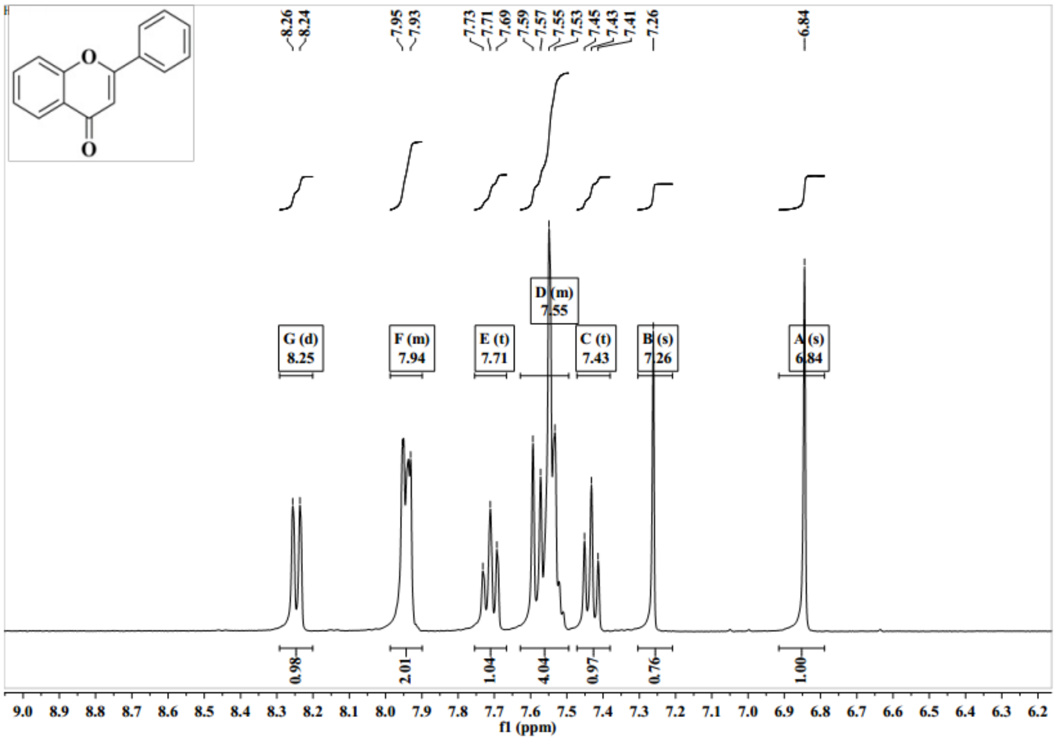

O
O
8.26
8.24
7.95
7.93
7.73
7.71
7.69
7.59
7.57
7.55
7.53
7.45
7.43
7.41
7.26
6.84
G (d)
8.25
F (m)
7.94
E (t)
7.71
D (m)
7.55
C (t)
7.43
B (s)
7.26
A (s)
6.84
0.98
2.01
1.04
4.04
0.97
0.76
1.00
9.0 8.9 8.8 8.7 8.6 8.5 8.4 8.3 8.2 8.1 8.0 7.9 7.8 7.7 7.6 7.5 7.4 7.3 7.2 7.1 7.0 6.9 6.8 6.7 6.6 6.5 6.4 6.3 6.2
f1 (ppm)

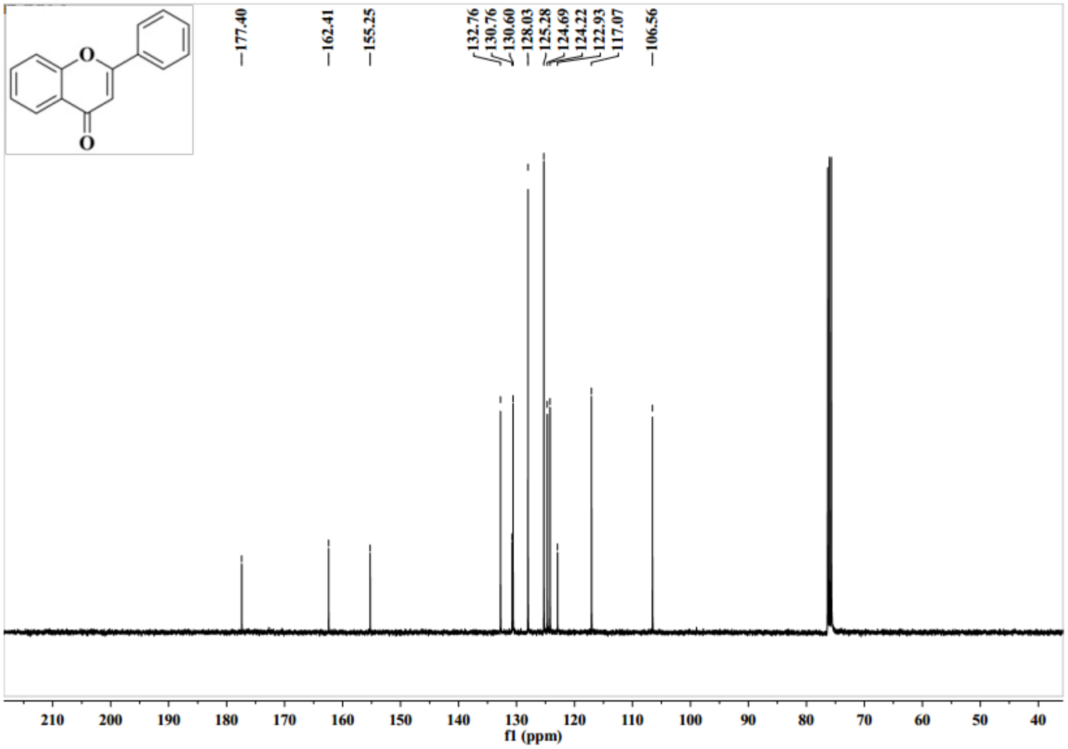

O
O
177.40
162.41
155.25
132.76
130.76
130.60
128.03
125.28
124.69
124.22
122.93
117.07
106.56
210 200 190 180 170 160 150 140 130 120 110 100 90 80 70 60 50 40
f1 (ppm)

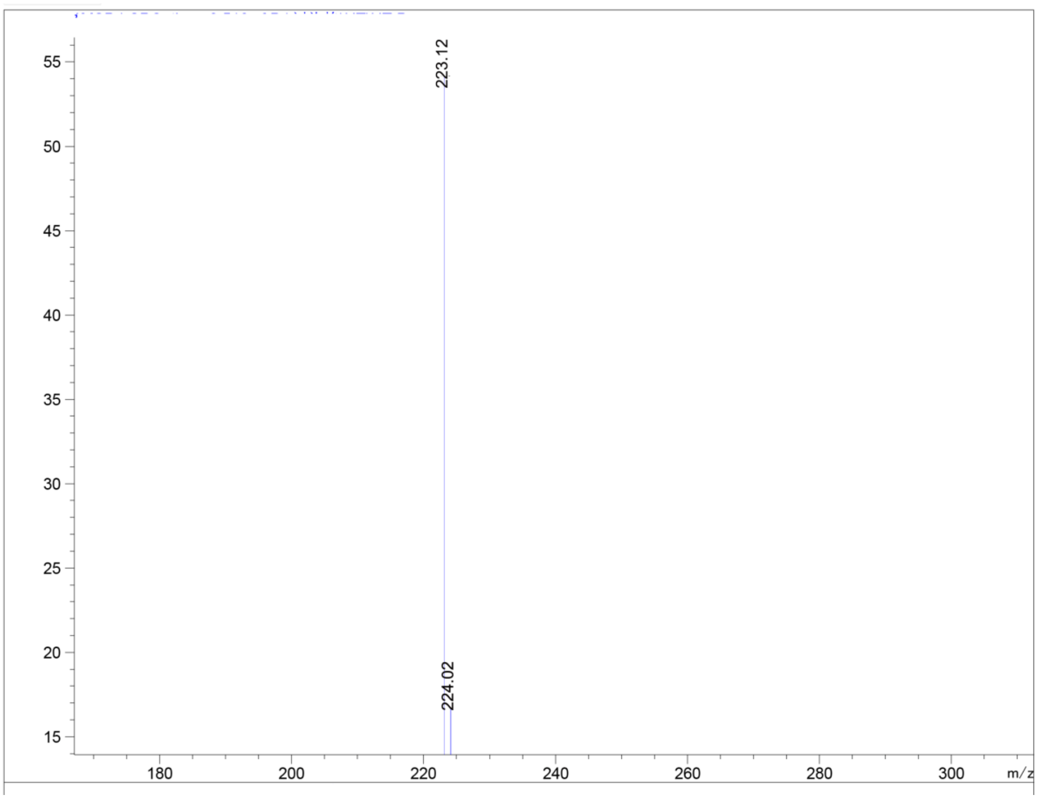

Figure S11.^{1}H, ^{13}C NMR spectra andESI-MS analysis for compound **5g.**

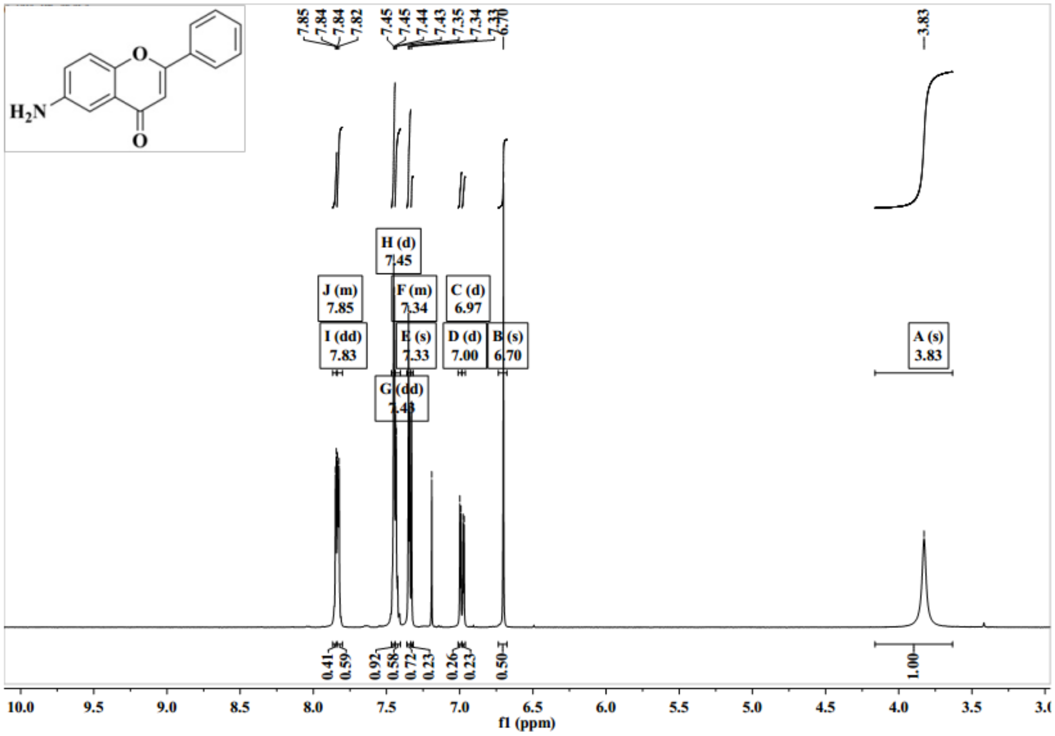

H$_2$N
O
7.85
7.84
7.84
7.82
7.45
7.45
7.44
7.43
7.35
7.34
7.33
6.70
3.83
H (d)
7.45
J (m)
7.85
F (m)
7.34
C (d)
6.97
I (dd)
7.83
E (s)
7.33
D (d)
7.00
B (s)
6.70
A (s)
3.83
G (dd)
7.43
0.41
0.59
0.92
0.58
0.72
0.23
0.26
0.23
0.50
1.00
10.0
9.5
9.0
8.5
8.0
7.5
7.0
6.5
6.0
5.5
5.0
4.5
4.0
3.5
f1 (ppm)

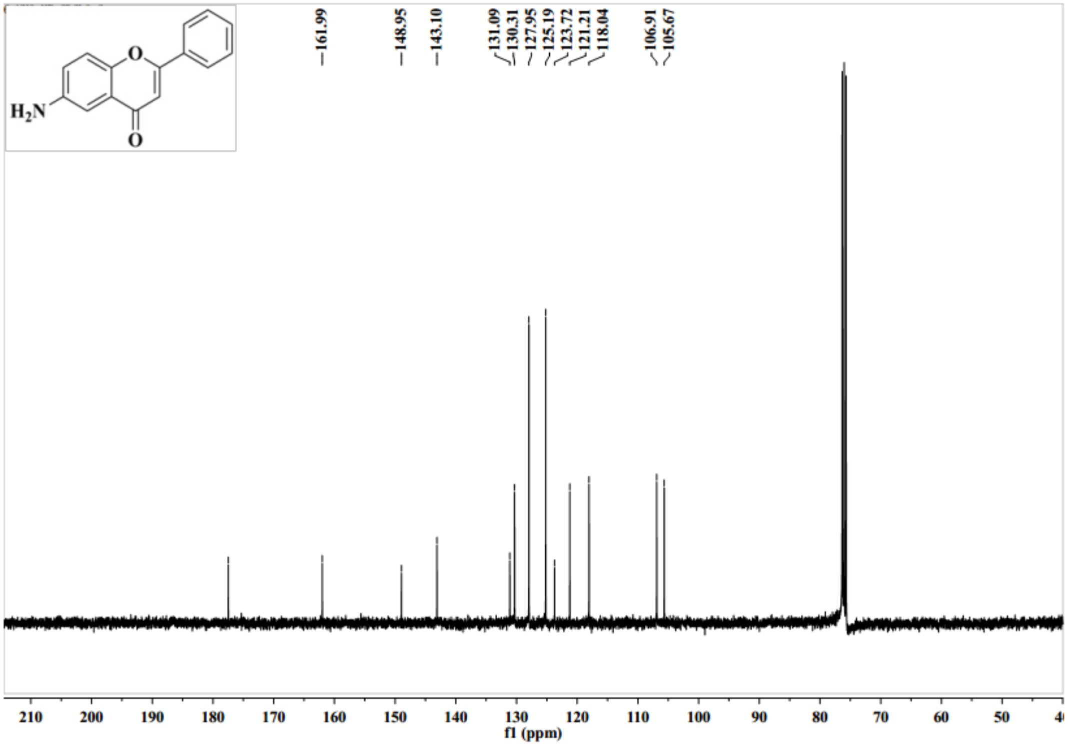

H$_2$N
O
161.99
148.95
143.10
131.09
130.31
127.95
125.19
123.72
121.21
118.04
106.91
105.67
210
200
190
180
170
160
150
140
130
120
110
100
90
80
70
60
50
f1 (ppm)

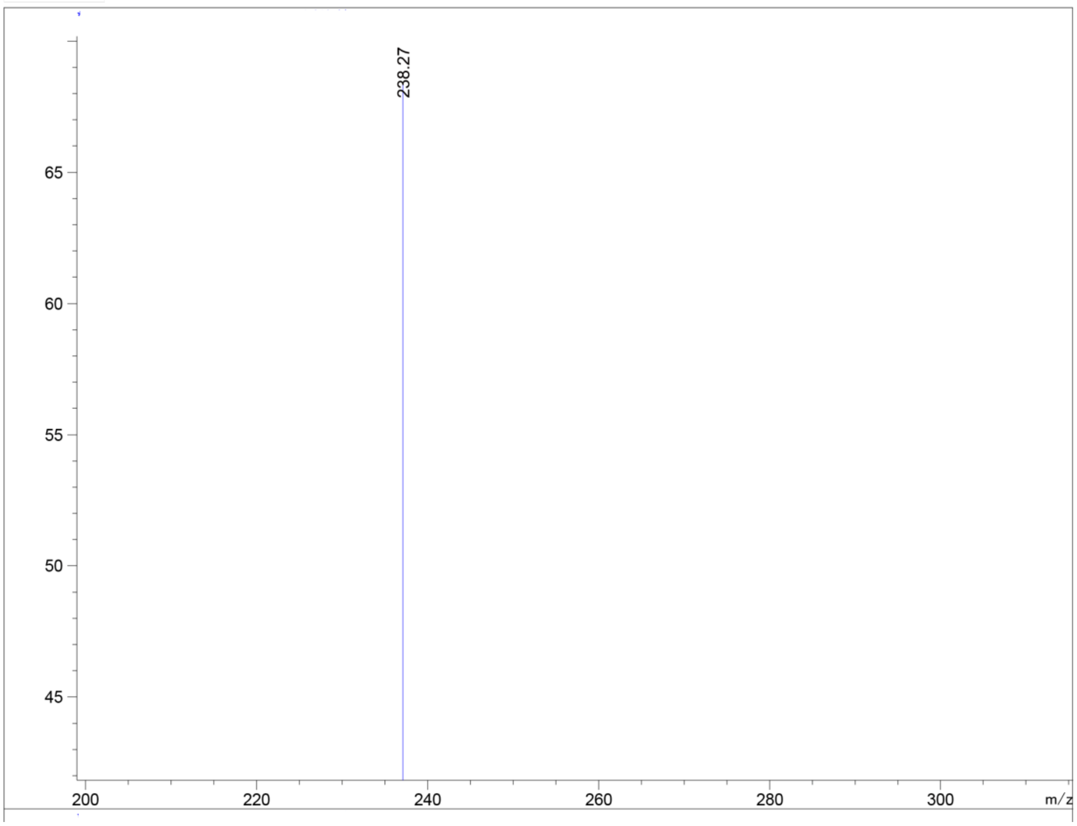

Figure S12.^{1}H, ^{13}C NMR spectra and ESI-MS analysis for compound **5h.**

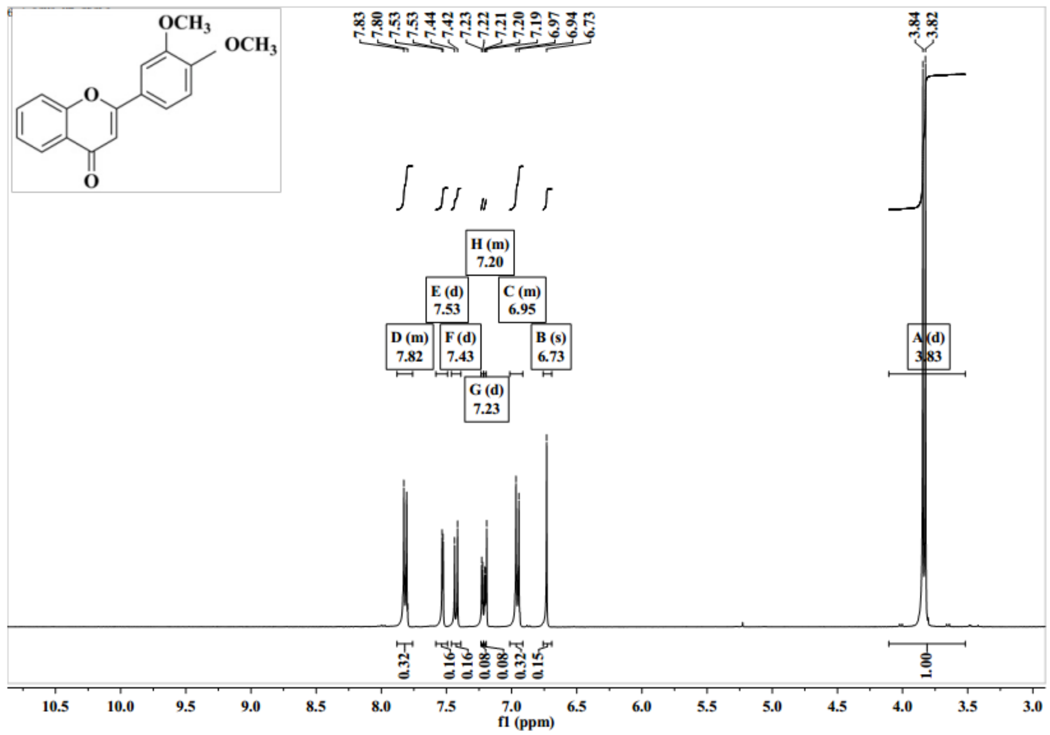

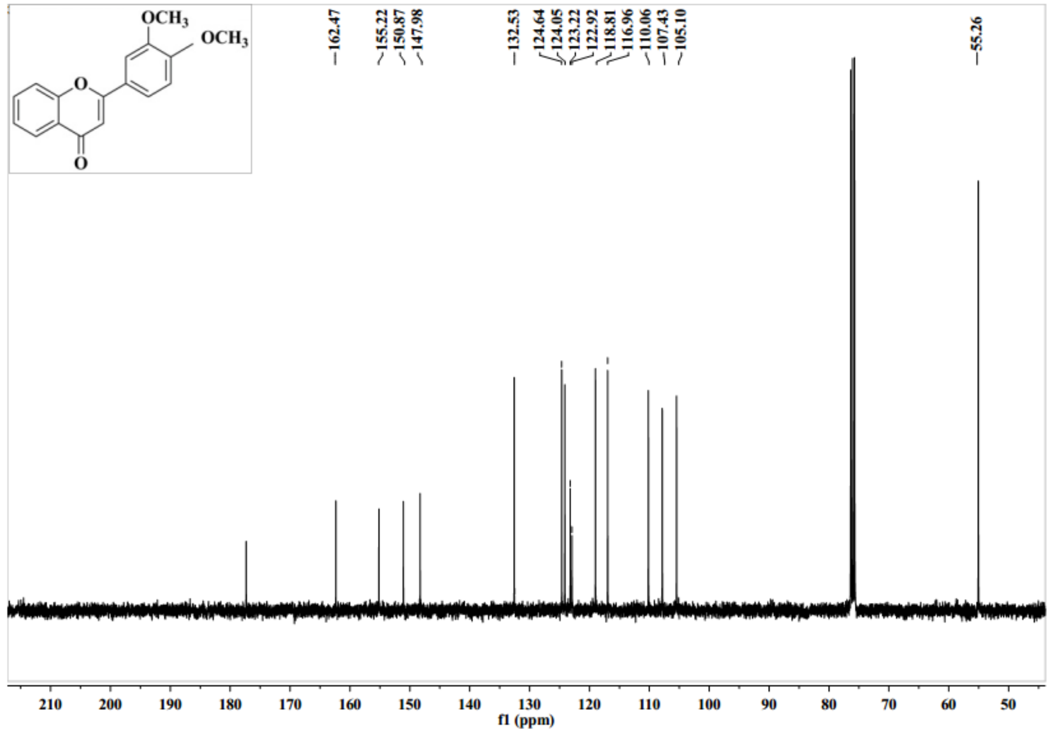

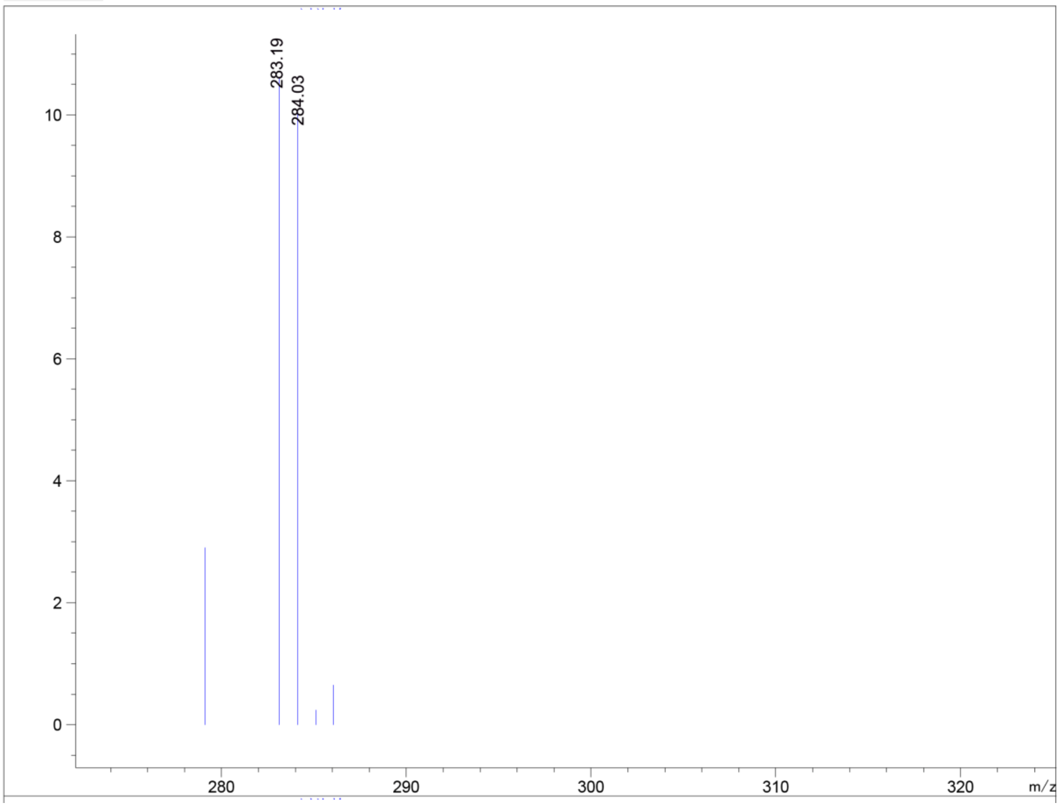

Figure S13.^{1}H, ^{13}C NMR spectra and ESI-MS analysis for compound **5i.**

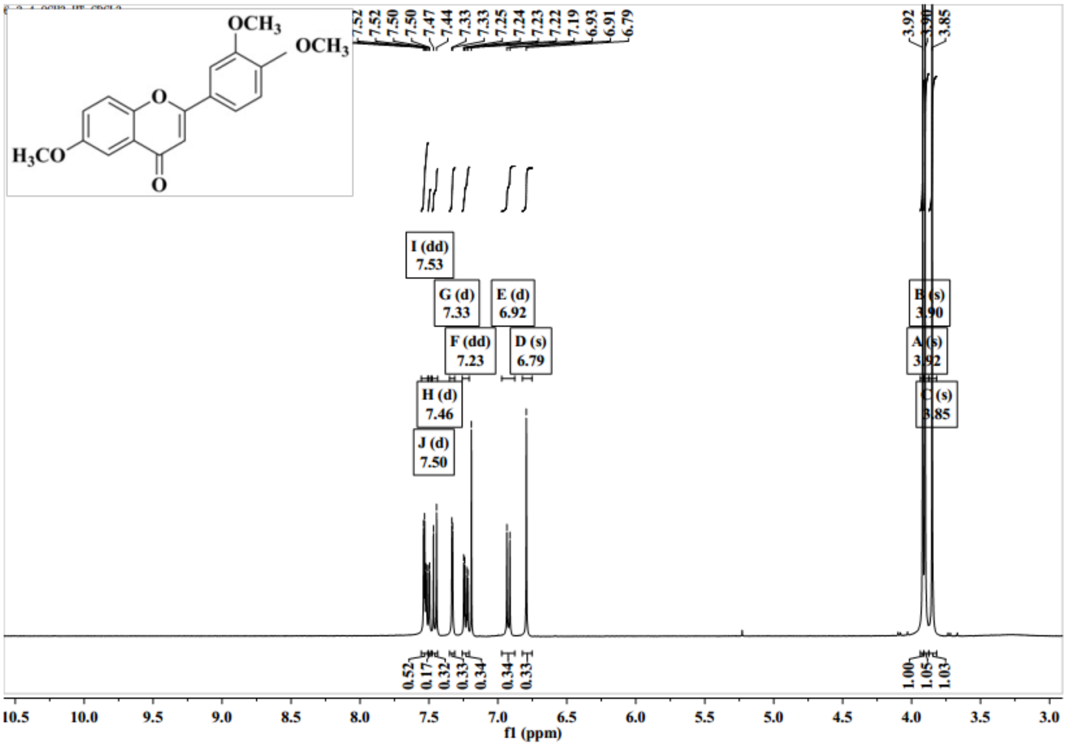
OCH3
OCH3
H3CO
O
I (dd)
7.53
G (d)
7.33
E (d)
6.92
F (dd)
7.23
D (s)
6.79
H (d)
7.46
J (d)
7.50
B (s)
3.90
A (s)
3.92
C (s)
3.85
f1 (ppm)

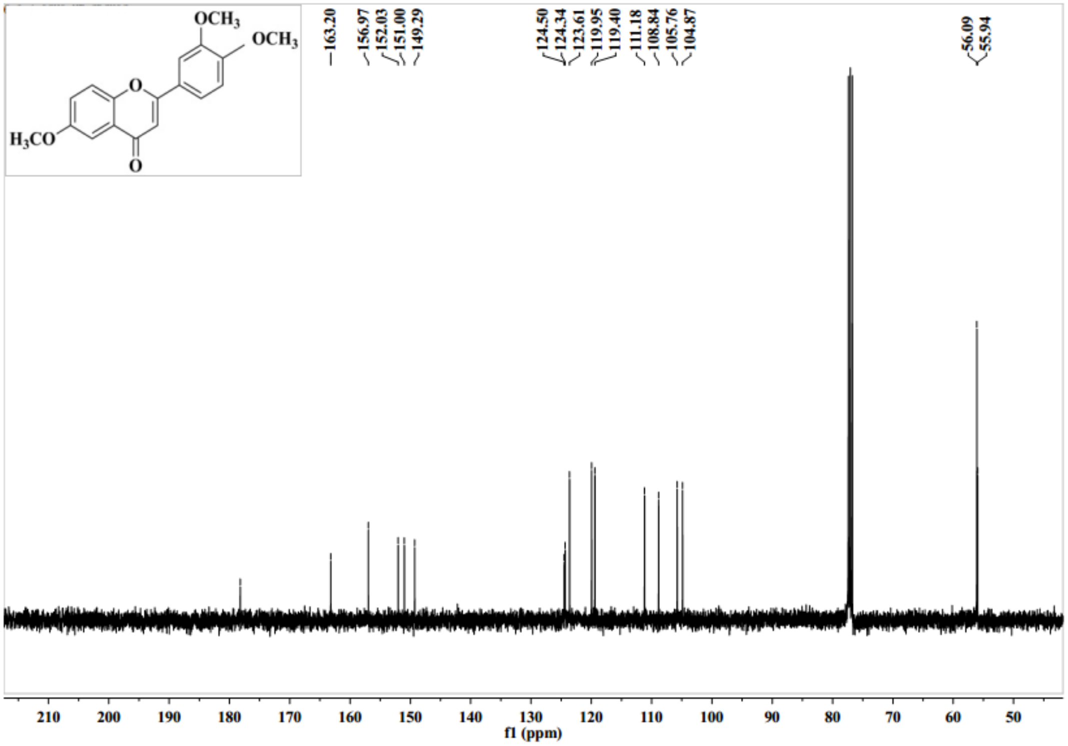
OCH3
OCH3
H3CO
O
163.20
156.97
152.03
151.00
149.29
124.50
124.34
123.61
119.95
119.40
111.18
108.84
105.76
104.87
56.09
55.94
f1 (ppm)

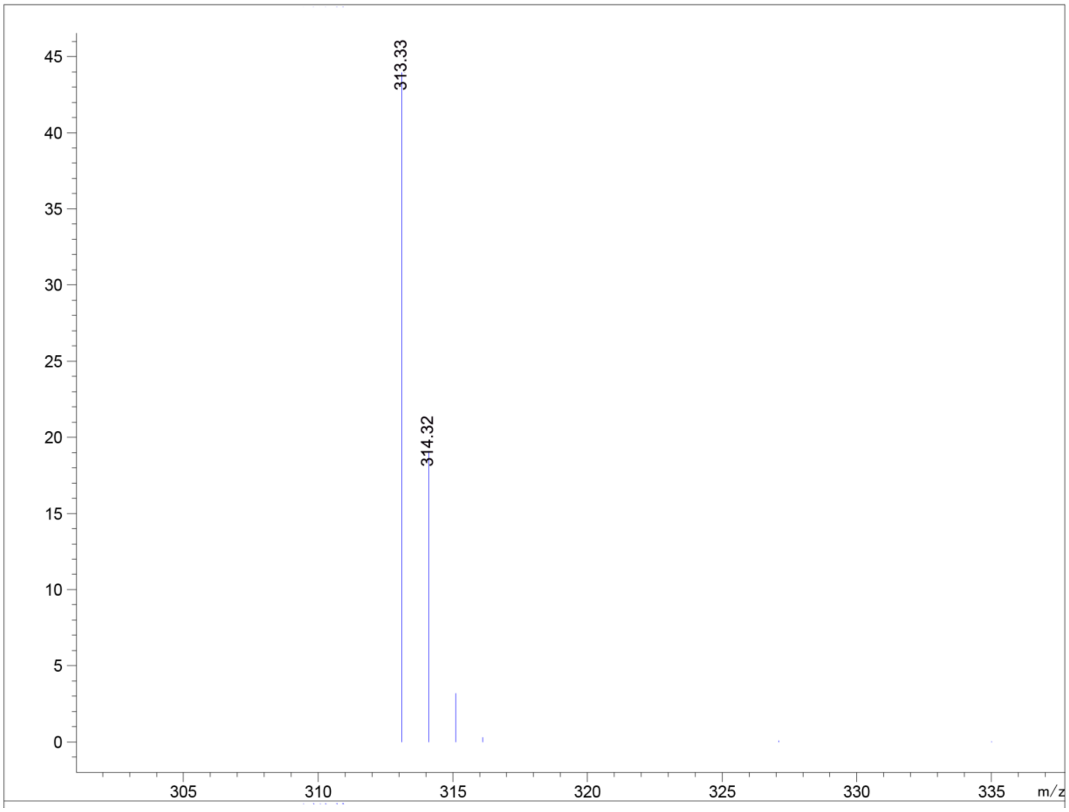

Figure S14. ^{1}H, ^{13}C NMR spectra and ESI-MS analysis for compound **5j.**
